# Th1 polarization defines the synovial fluid T cell compartment in oligoarticular juvenile idiopathic arthritis

**DOI:** 10.1172/jci.insight.149185

**Published:** 2021-09-22

**Authors:** Amélie M. Julé, Kacie J. Hoyt, Kevin Wei, Maria Gutierrez-Arcelus, Maria L. Taylor, Julie Ng, James A. Lederer, Siobhan M. Case, Margaret H. Chang, Ezra M. Cohen, Fatma Dedeoglu, Melissa M. Hazen, Jonathan S. Hausmann, Olha Halyabar, Erin Janssen, Jeffrey Lo, Mindy S. Lo, Esra Meidan, Jordan E. Roberts, Mary Beth F. Son, Robert P. Sundel, Pui Y. Lee, Talal Chatila, Peter A. Nigrovic, Lauren A. Henderson

**Affiliations:** 1 Division of Immunology, Boston Children’s Hospital, Harvard Medical School, Boston, Massachusetts, USA.; 2 Division of Rheumatology, Inflammation, and Immunity, Brigham and Women’s Hospital, Harvard Medical School, Boston, Massachusetts, USA.; 3 Broad Institute of MIT and Harvard, Cambridge, Massachusetts, USA.; 4 Division of Pulmonary and Critical Care Medicine, and; 5 Department of Surgery, Brigham and Women’s Hospital, Harvard Medical School, Boston, Massachusetts, USA.

**Keywords:** Autoimmunity, Immunology, Rheumatology, T cells, Th1 response

## Abstract

Oligoarticular juvenile idiopathic arthritis (oligo JIA) is the most common form of chronic inflammatory arthritis in children, yet the cause of this disease remains unknown. To understand immune responses in oligo JIA, we immunophenotyped synovial fluid T cells with flow cytometry, bulk RNA-Seq, single-cell RNA-Seq (scRNA-Seq), DNA methylation studies, and Treg suppression assays. In synovial fluid, CD4^+^, CD8^+^, and γδ T cells expressed Th1-related markers, whereas Th17 cells were not enriched. Th1 skewing was prominent in CD4^+^ T cells, including Tregs, and was associated with severe disease. Transcriptomic studies confirmed a Th1 signature in CD4^+^ T cells from synovial fluid. The regulatory gene expression signature was preserved in Tregs, even those exhibiting Th1 polarization. These Th1-like Tregs maintained Treg-specific methylation patterns and suppressive function, supporting the stability of this Treg population in the joint. Although synovial fluid CD4^+^ T cells displayed an overall Th1 phenotype, scRNA-Seq uncovered heterogeneous effector and regulatory subpopulations, including IFN-induced Tregs, peripheral helper T cells, and cytotoxic CD4^+^ T cells. In conclusion, oligo JIA is characterized by Th1 polarization that encompasses Tregs but does not compromise their regulatory identity. Targeting Th1-driven inflammation and augmenting Treg function may represent important therapeutic approaches in oligo JIA.

## Graphical abstract

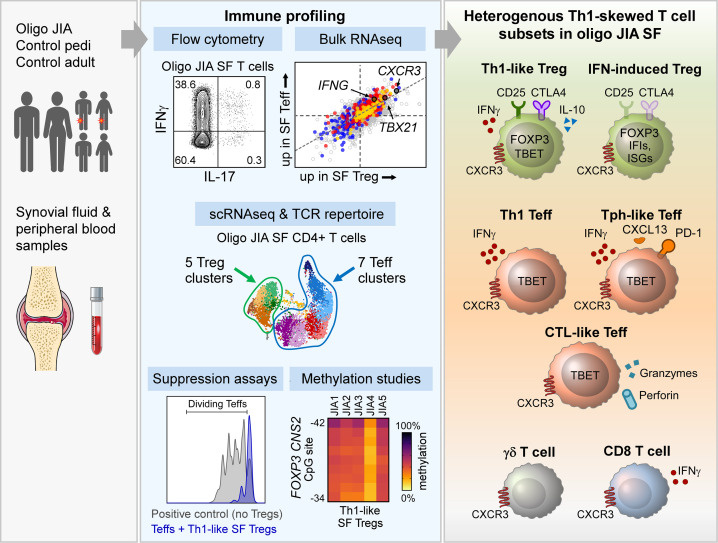

## Introduction

Chronic inflammatory arthritis in children is often considered a single disease by those outside the field of pediatric rheumatology. In fact, juvenile idiopathic arthritis (JIA) is an umbrella term that encompasses 6 disease onset types as defined by the International League of Associations for Rheumatology (ILAR) criteria (1). HLA associations and clinical characteristics further demonstrate that the term JIA includes several distinct diseases, whose underlying biological mechanisms are likely to differ (2, 3). Despite this heterogeneity, studies evaluating JIA have often included children with multiple forms of inflammatory arthritis without stratifying by disease type. We sought to focus on the pathogenesis of oligoarthritis, the most common form of JIA in North America (4).

Oligoarticular (oligo) JIA is defined by unique features that include limited joint involvement (<5 joints in the first 6 months of disease) and high rates of uveitis (1, 5). In some affected children, chronic arthritis remains restricted to a few joints (persistent oligo JIA), whereas in others it progresses to involve 5 or more joints (extended oligo JIA) (6). Given the limited number of arthritic joints at disease onset, children with oligo JIA are often initially treated with local intraarticular steroid injections instead of systemic immunosuppression. Thus, synovial fluid (SF) samples are easily accessible in this disease, offering an important opportunity to study pediatric autoimmunity at the site of inflammation.

In oligo JIA, the mechanisms that drive arthritis and result in a persistent or extended disease course are poorly understood. Prior work has uncovered inflammatory CD4^+^ effector T cells (Teffs) in oligo JIA joints. In studies that grouped oligoarthritis with other forms of JIA, Teffs with a Th17 phenotype were identified in the SF, and an inverse relationship between Th17 cells and Tregs was found (7, 8). An increased frequency of SF IL-17^+^CD4^+^ T cells in oligo JIA has been associated with disease extension (7). In studies restricted to oligo JIA and the closely related seronegative polyarticular JIA, T cells identified in the SF had predominantly Th1 features, including expression of IFN-γ, which is traditionally considered a Th1 cytokine (9–12). While IFN-γ^+^CD4^+^ T cells in oligo JIA SF may be bona fide Th1 cells differentiated from naive CD4^+^ T cells, an alternative hypothesis proposes that these cells are nonclassical Th1 cells derived from Th17 cells (13–15). Nonclassical Th1 cells coexpress Th1 and Th17 markers with respect to cytokines (IFN-γ, IL-17), chemokine receptors (CXCR3, CCR6), and transcription factors (T-bet, RORγt) (16, 17). Taken together, these past studies highlight the remaining uncertainty over what T cell populations, Th1, Th17, and/or nonclassical Th1 cells, are implicated pathogenically in oligo JIA.

The interplay between Teffs and Tregs, a subset of CD4^+^ T cells with suppressive capacity, represents another major area of interest in oligo JIA. In children with JIA, clonal Tregs are found in the SF, suggesting this T cell subset modulates immune responses in the arthritic joint (18, 19). Enrichment of Tregs in the SF is associated with fewer cytokine-expressing Teffs and a favorable disease course in oligo JIA (7, 20). Yet, Teffs that escape Treg-mediated suppression have been described in JIA (21–23). Further, the stability of Tregs in the inflammatory environment of the JIA joint is uncertain. Indeed, a proportion of Tregs in JIA SF express low levels of FOXP3, the transcription factor defining the Treg lineage (24). These findings indicate that the dynamics between Tregs and Teffs, as well as the stability of Tregs in the arthritic joint, are likely key factors that determine disease course in oligo JIA. Yet, the mechanisms by which proinflammatory and regulatory T cells influence the establishment and progression of arthritis in oligo JIA remain poorly understood.

Herein, we sought to determine the immunophenotype of joint-infiltrating CD4^+^ T cells as well as the stability of Tregs in oligo JIA. We showed that CD4^+^ T cells in the SF, including Tregs, were predominantly Th1 polarized. Disease extension was associated with a greater proportion of IFN-γ–expressing CD4^+^ T cells in the joint. Importantly, Th1-skewed SF Tregs in oligo JIA maintained Treg identity and suppressive functionality. Our findings suggest that inhibiting the Th1 pathway and enhancing Treg function may be key in controlling arthritic flares in oligo JIA.

## Results

### Patients and samples.

A total of 36 patients with oligo JIA, defined by ILAR criteria, provided SF and peripheral blood (PB) samples (Table 1). PB was also obtained from 8 pediatric and 10 adult controls. Details about the samples and experiments they contributed to are provided in Supplemental Table 1 (supplemental material available online with this article; https://doi.org/10.1172/jci.insight.149185DS1).

### CD4^+^ T cells in oligo JIA SF adopt a Th1 phenotype.

**Figure 1:**
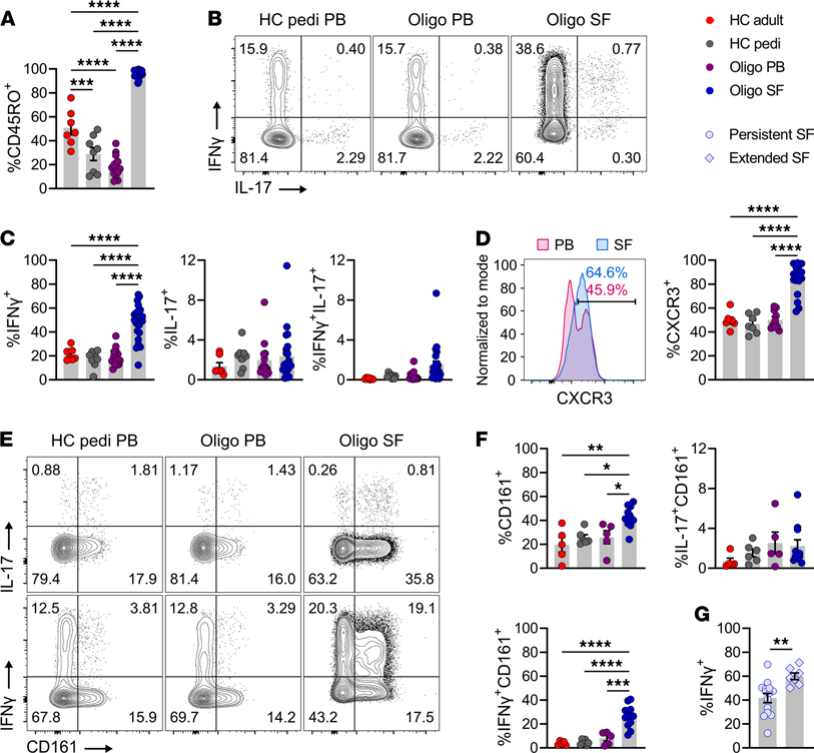
CD4^+^ memory T cells adopt a Th1 phenotype in the SF of oligo JIA. (**A**) Percentage of CD45RO^+^ cells among CD3^+^CD4^+^ lymphocytes. (**B**) Representative flow staining of cytokine production in stimulated CD3^+^CD4^+^CD45RO^+^ cells (CD4^+^ Tmem). (**C**) Percentage of CD4^+^ Tmem cells expressing IFN-γ, IL-17, or both after stimulation. The PB of adult (*n* = 7) and pediatric (*n* = 8) controls and PB (*n* = 14) and SF (*n* = 23) of oligo JIA patients were evaluated in **A** and **C**. (**D**) Representative histogram of CXCR3 MFI in paired PB and SF samples from an oligo JIA patient and quantification of CXCR3^+^ cells among unstimulated CD4^+^ Tmem cells from HC adult PB (*n* = 7), HC pediatric PB (*n* = 7), oligo JIA PB (*n* = 10), and SF (*n* = 17). (**E**) Representative flow staining of CD161 and cytokine production in stimulated cells gated on CD4^+^ Tmem cells. (**F**) Percentage of CD161^+^ cells (unstimulated) and of CD161^+^ and cytokine dual-expressing cells (stimulated) among CD4^+^ Tmem cells from HC adult PB (*n* = 5), HC pediatric PB (*n* = 6), oligo JIA PB (*n* = 5), and SF (*n* = 11). (**G**) Percentage of CD4^+^ Tmem cells expressing IFN-γ in SF samples from persistent (*n* = 14) and extended (*n* = 7) oligo JIA patients. Summary data on bar graphs are mean ± SEM. *P < 0.05, **P < 0.01, ***P < 0.001, ****P < 0.0001. Statistical testing: (**A**–**F**) 1-way ANOVA followed by multiple 2-tailed *t* tests with Tukey’s correction; (**G**) 2-tailed *t* test. HC, healthy control; oligo, oligoarticular juvenile idiopathic arthritis; pedi, pediatric; PB, peripheral blood; SF, synovial fluid.

Flow cytometry was used to evaluate the T cell compartment in oligo JIA. Memory CD4^+^, memory CD8^+^, and γδ T cells were enriched in oligo JIA joints (Figure 1A and Supplemental Figure 1, A and B). The frequencies of CD8^+^ memory T (Tmem) cells expressing the Th1 cytokine (IFN-γ) and chemokine receptor (CXCR3) were increased in oligo JIA SF compared with control PB (Supplemental Figure 1A). Similarly, the proportion of γδ T lymphocytes expressing CXCR3 was higher in SF samples (Supplemental Figure 1B). SF CD4^+^ Tmem cells showed the most pronounced upregulation of both IFN-γ and CXCR3 (Figure 1). On average, 47.8% ± 3.1% (mean ± SEM) and 83.9% ± 3.2% of CD4^+^ Tmem cells in SF expressed IFN-γ and CXCR3, respectively (Figure 1, B–D). Since intracellular cytokine detection requires stimulation, which leads to CXCR3 downregulation, we were unable to assess for CXCR3^+^IFN-γ^+^ cells. The frequency of CD4^+^ Tmem, CD8^+^ Tmem, and γδ T cells expressing IL-17 was not increased in the SF compared with the PB (Figure 1, B and C, and Supplemental Figure 1). These results highlight Th1 skewing across T lymphocyte subsets in oligo JIA SF, particularly in CD4^+^ T cells.

We characterized CD4^+^ Tmem cells with additional flow cytometry studies. Paired PB and SF samples confirmed enrichment of CXCR3^+^ and IFN-γ^+^CD4^+^ Tmem cells in oligo JIA joints (Supplemental Figure 2A). To assess for nonclassical Th1 cells that jointly express Th1 and Th17 features, we evaluated the fraction of CD4^+^ T cells producing IFN-γ and IL-17. This population was small (<2% of CD4^+^ Tmem in the joint on average) and not significantly increased in JIA SF compared with healthy control PB (Figure 1, B and C). In patients with paired samples, IFN-γ^+^IL-17^+^CD4^+^ Tmem cells were slightly more frequent in the SF (1.2% ± 0.4% of CD4^+^ Tmem) than in the PB (0.5% ± 0.19%) (Supplemental Figure 2A). T cell stimulation alters expression of the Th17-associated chemokine receptor, CCR6; therefore, CD161 was used as an alternative marker of Th17 cells (25). The SF of oligo JIA patients was moderately enriched in CD161^+^CD4^+^ Tmem cells but at a frequency lower than CXCR3^+^CD4^+^ Tmem cells (Figure 1, D–F, and Supplemental Figure 2). IFN-γ^+^CD161^+^CD4^+^ Tmem cells were enriched in the joint, whereas IL-17^+^CD161^+^CD4^+^ Tmem cells were not (Figure 1, E and F, and Supplemental Figure 2A). Overall, a large proportion of CD4^+^ Tmem cells in oligo JIA SF expressed IFN-γ without IL-17, and over half of CXCR3^+^ cells in the joint did not coexpress CD161, suggesting a substantial population of classical Th1 cells in the joint.

The fraction of IFN-γ^+^CD4^+^ Tmem cells was higher in the SF of patients with extended (59.9% ± 2.8%) versus persistent (41.6% ± 3.9%) oligo JIA (Figure 1G). The frequency of IL-17^+^, IFN-γ^+^IL-17^+^, CXCR3^+^, CD161^+^, and IFN-γ^+^CD161^+^ CD4^+^ Tmem cells did not significantly differ between these 2 groups (Supplemental Figure 2C). The presence of autoantibodies (antinuclear antibody [ANA] positivity) was not associated with differences in the frequency of these T cell populations (Supplemental Figure 2D). SF sampled at disease onset or during a flare of chronic disease showed similar proportions of CD4^+^ Tmem cells expressing CXCR3 and IFN-γ (Supplemental Figure 2B). Therefore, the Th1 predominance found in oligo JIA SF persisted throughout the disease course and was associated with severe disease.

### CD4^+^ Tregs in oligo JIA SF express Th1 markers.

**Figure 2:**
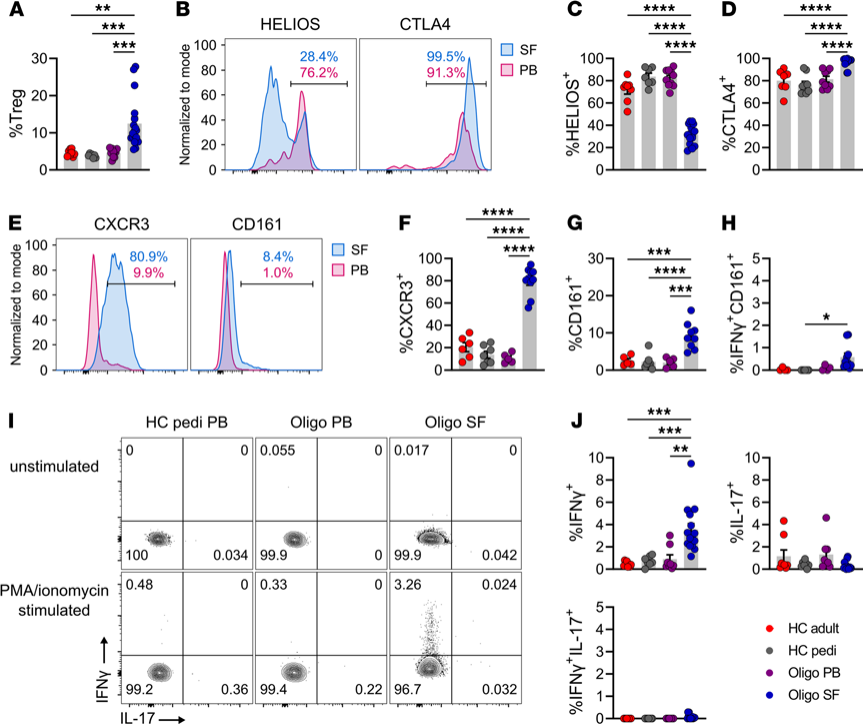
The SF in oligo JIA patients is enriched in Tregs expressing Th1 markers. (**A**) Percentage CD25^+^CD127^lo^FOPX3^+^ (Tregs) cells among CD4^+^ lymphocytes in the PB of adult (*n* = 8) and pediatric (*n* = 7) controls and in the PB (*n* = 10) or SF (*n* = 17) of oligo JIA patients. (**B**) Representative histogram of HELIOS (unstimulated cells) and CTLA4 (stimulated cells) MFI in paired PB and SF samples from an oligo JIA patient, gated on Tregs. (**C**) Percentage Tregs expressing HELIOS in HC adult PB (*n* = 8), HC pediatric PB (*n* = 7), oligo JIA PB (*n* = 10), and SF (*n* = 15). (**D**) Percentage Tregs expressing CTLA4 after stimulation in HC adult PB (*n* = 7), HC pediatric PB (*n* = 9), oligo JIA PB (*n* = 9), and SF (*n* = 16). (**E**) Representative histogram of CXCR3 and CD161 MFI in unstimulated Tregs. (**F**) Percentage Tregs expressing CXCR3 in HC adult PB (*n* = 6), HC pediatric PB (*n* = 7), oligo JIA PB (*n* = 5), and SF (*n* = 10). (**G**) Percentage Tregs expressing CD161 in HC adult PB (*n* = 5), HC pediatric PB (*n* = 7), oligo JIA PB (*n* = 5), and SF (*n* = 9). (**H**) Percentage Tregs jointly expressing IFN-γ and CD161 after stimulation in HC adult PB (*n* = 4), HC pediatric PB (*n* = 7), oligo JIA PB (*n* = 4), and SF (*n* = 10). (**I**) Representative flow staining of cytokine production in unstimulated and stimulated Tregs. (**J**) Percentage of Tregs expressing IFN-γ, IL-17, or both cytokines after stimulation in HC adult PB (*n* = 8), HC pediatric PB (*n* = 7), oligo JIA PB (*n* = 8), and SF (*n* = 16). Summary data on bar graphs are mean ± SEM. *P < 0.05, **P < 0.01, ***P < 0.001, ****P < 0.0001. Statistical testing: 1-way ANOVA followed by multiple 2-tailed *t* tests with Tukey’s correction. HC, healthy control; pedi, pediatric; oligo, oligoarticular juvenile idiopathic arthritis; PB, peripheral blood; SF, synovial fluid.

As reported previously, an increased frequency of CD4^+^CD25^+^CD127^lo^FOXP3^+^ cells was observed in oligo JIA SF compared with PB (Figure 2A and Supplemental Figure 3A) (20, 22). The SF Treg compartment was depleted in HELIOS^+^ cells, a putative marker of thymically derived Tregs, and SF Tregs expressed high levels of CTLA4 (Figure 2, B–D, and Supplemental Figure 3A) (26, 27). A majority of SF Tregs were CXCR3^+^ (79.9% ± 3.9%), in stark contrast with PB Tregs (Figure 2, E and F, and Supplemental Figure 3A). Significantly higher frequencies of CD161^+^ Tregs were found in the SF than in the PB (Figure 2, E–G, and Supplemental Figure 3A), but compared with CXCR3^+^ Tregs, the proportion of CD161^+^ Tregs (9.2% ± 1.2%) remained low in the joint. Upon stimulation, 3.6% ± 0.5% of SF Tregs secreted IFN-γ, a significant increase compared with Tregs from the PB of patients (0.9% ± 0.4%) and pediatric (0.73% ± 0.17%) and adult (0.46% ± 0.08%) controls (Figure 2, I and J, and Supplemental Figure 3). IL-17–expressing Tregs, which have been identified in systemic JIA, were not found in oligo JIA (28). CXCR3^+^ and IFN-γ^+^ Tregs were found at similar levels in the SF of oligo JIA patients with new-onset and chronic disease, regardless of ANA positivity and disease course (Supplemental Figure 3, B–D). Overall, as in CD4^+^ Tmem cells, Tregs with predominantly Th1 features (Th1-like Tregs) were found in oligo JIA SF.

### CD4^+^ T cells in oligo JIA SF express a Th1 transcriptomic signature.

**Figure 3:**
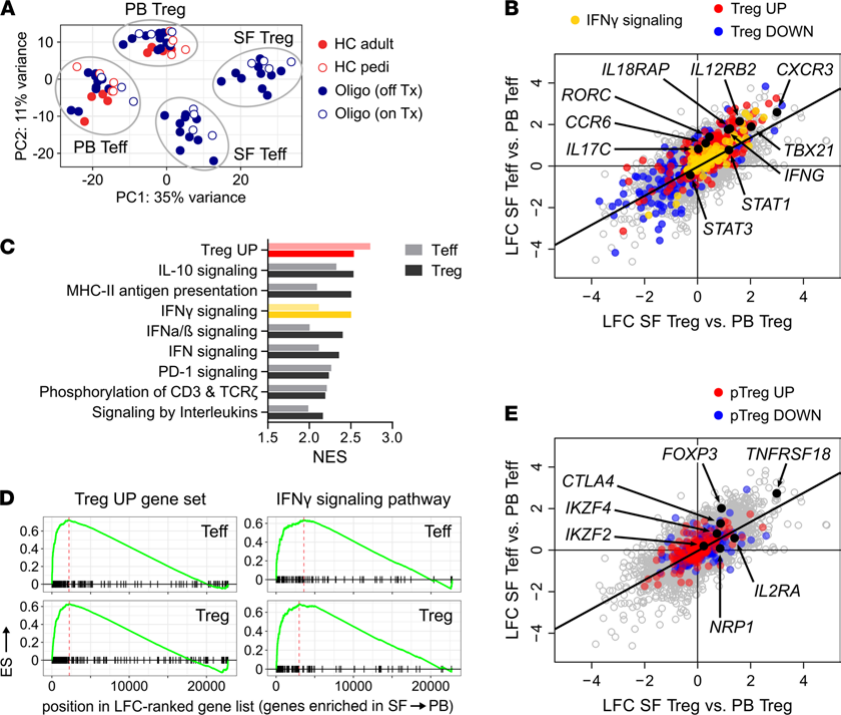
Teffs and Tregs in oligo JIA SF upregulate IFN-γ–related genes. Tregs (CD4^+^CD25^+^CD127^lo^) and Teffs (CD4^+^CD25^–^) were sorted from paired SF and PB samples of JIA patients (*n* = 14) and from the PB of pediatric (*n* = 5) and adult (*n* = 4) controls. (**A**) Principal component (PC) plot based on analysis of complete transcriptomes. (**B**) Log_2_ fold change (LFC) in gene expression derived from independent differential gene expression analyses of SF Tregs versus PB Tregs (*x* axis) and SF Teffs versus PB Teffs (*y* axis). Genes of the IFN-γ signaling pathway (Reactome database) and Treg signature (30) are color-coded. Selected Th1- and Th17-related genes are annotated. (**C**) Normalized enrichment score (NES) of top hits in gene set enrichment analyses (GSEAs) of Treg and Teff subsets (see Supplemental Table 4 for complete GSEA results). (**D**) Running enrichment scores for the set of genes upregulated in classical Tregs (30) and for the IFN-γ signaling pathway (Reactome database). (**E**) LFC in gene expression derived from differential gene expression analysis of Treg (*x* axis) and Teff (*y* axis) subsets. Genes of the peripherally induced Treg (pTreg) signature (29) are color-coded. Selected Treg-related genes are annotated. HC, healthy control; pedi, pediatric; oligo, oligoarticular juvenile idiopathic arthritis; Tx, treatment.

To further understand gene expression in CD4^+^ T cells in oligo JIA, Tregs and Teffs from patients and controls were assessed with bulk RNA-Seq (Figure 3; for interactive interface of transcriptomic results, see https://amjule.shinyapps.io/oligo-JIA/). Principal component analysis (PCA) of the transcriptomic data segregated samples by compartment (PB versus SF) and cell type (Teff versus Treg) (Figure 3A), even for patients receiving methotrexate.

Like flow cytometry analyses, differential gene expression analysis showed that SF Tregs and SF Teffs upregulated *IFNG* and *CXCR3* expression as well as *TBX21*, which encodes the Th1 lineage-defining transcription factor T-bet (Figure 3B and Supplemental Data File 1). Gene set enrichment analysis (GSEA) highlighted IFN-γ signaling as one of the top enriched gene sets in SF Tregs and SF Teffs compared with PB Tregs and PB Teffs, respectively (Figure 3, C and D). Gene sets related to antigen presentation, T cell receptor (TCR) signaling, and type I IFNs were also enriched in SF Tregs and SF Teffs (Figure 3C, Supplemental Table 4). Th17-related genes were not expressed in SF Tregs at levels significantly higher than in PB Tregs. Transcripts of the Th17 chemokine receptor *CCR6* and master gene *RORC* showed minor increases in SF Teffs compared with PB Teffs, but no differences in IL-17 expression were detected. These results confirm Th1 polarization at the transcriptional level in Tregs and Teffs from the oligo JIA joint.

### Oligo JIA SF Tregs maintain robust expression of Treg-related genes.

Flow cytometry uncovered cytokine-producing SF Tregs, indicating that these cells may have been reprogrammed to an effector population. To assess for this possibility, we evaluated the transcriptomic signature of Tregs in oligo JIA. Treg-associated transcripts remained significantly elevated in SF Tregs compared with PB Tregs (Figure 3E and Supplemental Data File 1). The Treg transcriptional signature was among the most enriched gene sets in SF Tregs (Figure 3, C and D). Tregs can be thymic in origin (tTreg) or generated in the periphery (pTreg). The low protein expression of HELIOS in SF Tregs documented by flow cytometry suggested pTreg enrichment in the joint; however, the pTreg signature was not found in the transcriptome of SF Tregs (Figure 3E and Supplemental Table 4) (29). Compared with circulating Teffs, SF Teffs upregulated some genes from the Treg transcriptional signature (Figure 3, B–D). This phenomenon is likely secondary to T cell activation and TCR stimulation, which can induce transient expression of some Treg-related genes (30–33). When SF Tregs were compared directly with SF Teffs, the Treg population retained the highest levels of regulatory gene expression, including *FOXP3* (Supplemental Data File 1). Thus, despite increased expression of Th1-related genes, SF Tregs evaluated as a bulk population maintained a regulatory transcriptional program.

### Th1-like Tregs in oligo JIA SF maintain features of functional Tregs.

**Figure 4:**
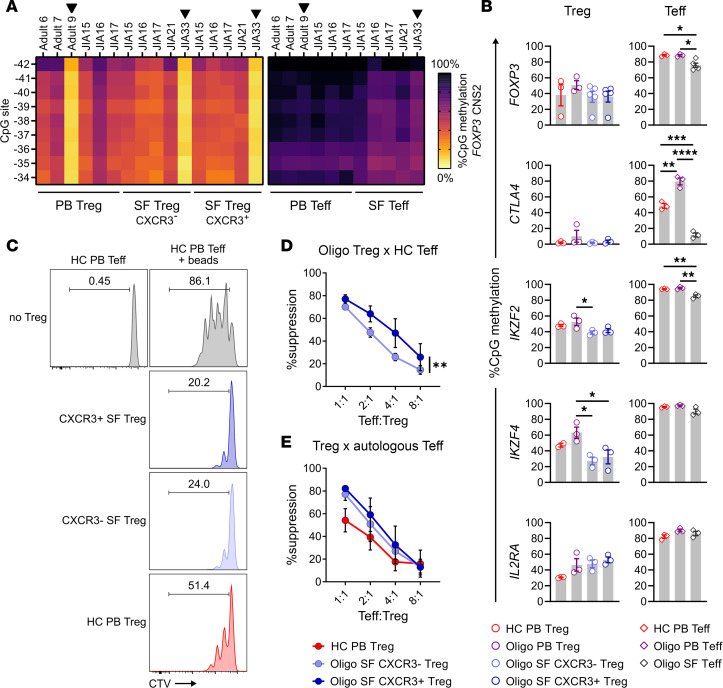
Th1-like Tregs maintain Treg-specific demethylation patterns and suppressive function. (**A**) Heatmap of methylation at 9 CpG sites in the conserved noncoding sequence 2 (CNS2) region of the *FOXP3* locus in sorted Tregs (CD4^+^CD25^+^CD127^lo^) and Teffs (CD4^+^CD25^–^) from HC adult PB (*n* = 3) and oligo JIA PB (*n* = 3) and SF (*n* = 5). Black arrows highlight male subjects. (**B**) Percentage methylation (mean ± SEM) at CpG sites across different Treg-related gene loci in sorted Treg and Teff populations. The location of CpG sites for each locus is specified in Supplemental Table 3. *IKZF2* encodes for HELIOS and *IKZF4* encodes for EOS. (**C**) Representative histogram of CTV-positive Teffs (CD4^+^CD25^–^) after 4 days of coculture alone or with anti-CD2/CD3/CD28 beads and the corresponding Treg (CD4^+^CD25^+^CD127^lo^) population at a ratio of 1:1, gated on dividing Teffs. (**D**) Percentage suppression (mean ± SEM) in the proliferation of Teffs from a third-party control after cocultures with CXCR3^+^ SF Tregs or CXCR3^–^ SF Tregs (*n* = 4 controls, *n* = 7 oligo JIA patients). See Supplemental Figure 4 for detailed representation per patient. (**E**) Percentage suppression (mean ± SEM) in the proliferation of Teffs from HC PB or oligo SF after cocultures with autologous Tregs (*n* = 4 controls, *n* = 4 oligo JIA patients). *P < 0.05, **P < 0.01, ***P < 0.001, ****P < 0.0001. Statistical testing: (**B**) 1-way ANOVA followed by multiple 2-tailed *t* tests with Tukey’s correction; (**D** and **E**) 2-way ANOVA assessing effect of Treg population and Teff/Treg ratio. HC, healthy control; oligo, oligoarticular juvenile idiopathic arthritis; PB, peripheral blood; SF, synovial fluid; CTV, CellTrace Violet.

We then sought to determine the stability and functionality of Th1-skewed (CXCR3^+^) SF Tregs through methylation studies and suppression assays (Figure 4). CXCR3^+^ SF Tregs maintained hypomethylation in the conserved noncoding sequence 2 (CNS2) of *FOXP3* and in other Treg-related loci (Figure 4, A and B), as expected for stable Tregs (34). Compared with PB Teffs, SF Teffs displayed significantly lower levels of methylation at *FOXP3*, *CTLA4*, and *IKZF2* loci, consistent with our transcriptomic findings and the known ability of TCR stimulation to induce epigenetic changes in these genes, even in non-Tregs (35).

Importantly, CXCR3^+^ and CXCR3^–^ SF Tregs demonstrated a preserved ability to suppress Teff proliferation and cytokine production (Figure 4, C–E, and Supplemental Figure 4). When assessed against Teffs isolated from the PB of different third-party controls, CXCR3^+^ SF Tregs demonstrated an enhanced ability to inhibit Teff proliferation as compared with CXCR3^–^ SF Tregs (Figure 4D and Supplemental Figure 4A). To determine whether SF Teffs are resistant to SF Treg–mediated suppression, autologous SF Teffs were cocultured with both CXCR3^+^ and CXCR3^–^ SF Treg subsets. Both CXCR3^+^ and CXCR3^–^ SF Tregs effectively inhibited SF Teff proliferation to a similar degree, which also did not differ from the suppressive capacity of control PB Tregs versus autologous PB Teffs (Figure 4E). Compared with SF Teffs cultured with anti-CD2/CD3/CD28 beads alone, the addition of CXCR3^+^ SF Tregs significantly decreased the levels of IFN-γ in the culture supernatants, indicating a preserved Treg capacity to inhibit cytokine production (Supplemental Figure 4B). Taken together, these bulk-level studies showed stable epigenetic imprinting of the Treg program and preserved suppressive capacity in CXCR3^+^ and CXCR3^–^ SF Tregs.

### Heterogeneity at the single-cell level in Th1-skewed CD4^+^ T cells in SF.

**Figure 5:**
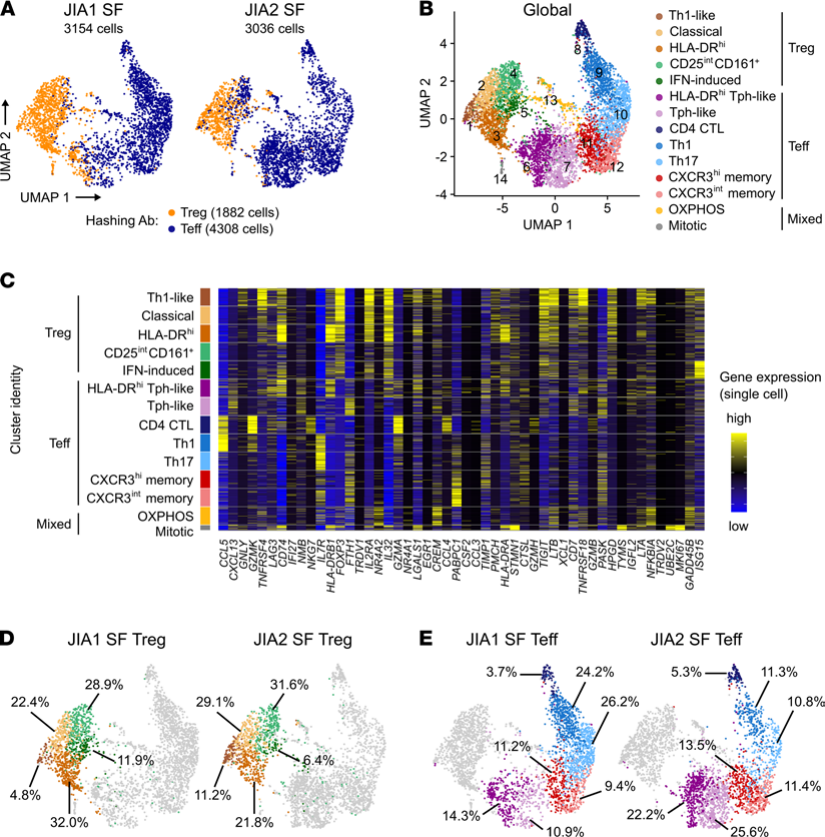
ScRNA-Seq reveals heterogeneous subsets of Tregs and Teffs in oligo JIA SF. Sorted Tregs (CD4^+^CD25^+^CD127^lo^) and Teffs (CD4^+^CD25^–^) from the SF of 2 oligo JIA patients were studied with 10x Genomics. (**A**) Uniform manifold approximation and projection (UMAP) of data set, split by patient and color-coded by hashing antibody. (**B**) UMAP of global data set (both subjects, 6190 cells), color-coded by cluster. (**C**) Heatmap showing expression of the top 50 most highly variable genes in 100 randomly selected cells from each cluster. (**D** and **E**) UMAP highlighting cells in Treg (**D**) and Teff (**E**) clusters, split by patient and annotated with the corresponding percentage of cells in each cluster. Clusters were defined as 1) Th1-like Treg, 2) classical Treg, 3) activated/HLA-DR^hi^ Treg, 4) CD25^int^CD161^+^ Treg, 5) IFN-induced Treg, 6) activated/HLA-DR^hi^ T peripheral helper–like (Tph-like), 7) Tph-like, 8) CD4^+^ cytotoxic T lymphocytes (CTLs), 9) Th1 effector memory, 10) Th17 effector memory, 11) CXCR3^hi^ central memory, 12) CXCR3^lo^ central memory, 13) cells undergoing OXPHOS metabolism, 14) mitotic cells. JIA, juvenile idiopathic arthritis; SF, synovial fluid.

**Figure 6:**
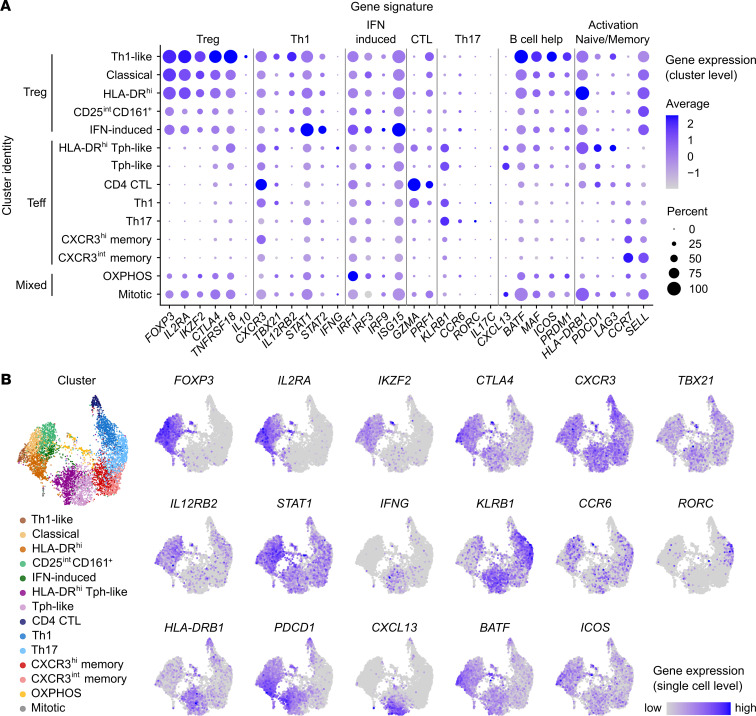
Gene expression in Th1-polarized Tregs and Teffs from oligo JIA SF. Sorted Tregs (CD4^+^CD25^+^CD127^lo^) and Teffs (CD4^+^CD25^–^) from the SF of 2 oligo JIA patients were studied with 10x Genomics. (**A**) Cluster-level expression of markers from key immune cell types (Treg, Th1, Th17, CTLs, Tph/B cell helper T cells), states (activation, exhaustion, naive/memory), and pathways (IFN signaling, viral sensing) used to characterize clusters. For each gene and cluster, the size of the dot is proportional to the percentage of cells expressing the gene in that cluster, and the color code illustrates the average level of expression of all cells in the cluster. (**B**) Expression levels of key Treg-, Th1-, Th17-, and Tph-related genes across the 6190 cells. The global UMAP color-coded by cluster is shown again for reference. For each gene, cells in the top 95% quantile of expression level are color-coded; the bottom 5% appear in gray. Additional genes can be browsed at https://amjule.shinyapps.io/oligo-JIA/ Clusters were defined as 1) Th1-like Treg, 2) classical Treg, 3) activated/HLA-DR^hi^ Treg, 4) CD25^int^CD161^+^ Treg, 5) IFN-induced Treg, 6) activated/HLA-DR^hi^ T peripheral helper–like (Tph-like), 7) Tph-like, 8) CD4^+^ cytotoxic T lymphocytes (CTLs), 9) Th1 effector memory, 10) Th17 effector memory, 11) CXCR3^hi^ central memory, 12) CXCR3^lo^ central memory, 13) cells undergoing OXPHOS metabolism, 14) mitotic cells.

Coexpression of Th1- and Th17-related genes in the same cell is a hallmark of nonclassical Th1 cells that bulk transcriptomic studies cannot unravel. Moreover, the robustness of the Treg transcriptomic signature in the subpopulation of Tregs with Th1 features cannot be determined from bulk RNA-Seq data. Thus, we conducted single-cell RNA-Seq (scRNA-Seq) and TCR repertoire analysis on sorted Tregs and Teffs from the SF of 2 oligo JIA patients. After quality control, the single-cell data set comprised 1882 Tregs and 4308 Teffs as defined by hashing antibody (Figure 5A). High-dimensional analysis uncovered 14 clusters, including 5 dominated by cells with a Treg profile (clusters 1 to 5; 2004 cells) and 7 dominated by Teffs (clusters 6 to 12; 3955 cells) (Figure 5, B–E; and Figure 6, A and B; https://amjule.shinyapps.io/oligo-JIA/). The final 2 clusters (231 cells) were best characterized by a mitochondrial (cluster 13) or mitotic (cluster 14) signature.

### Treg clusters.

Five SF Treg clusters were identified at comparable frequencies in both patients (Figure 5D). Key genes associated with the Treg (*FOXP3*, *IL2RA*, *IKZF2*, *CTLA4*, *TNFRSF18*) and Th1 (*CXCR3*, *TBX21*, *STAT1*, *IL12RB2*) profile were detected in all Treg clusters, with differential expression of these and cluster-defining genes across Treg subsets (Figures 5 and 6).

Cluster 1 exhibited robust expression of regulatory genes, including the highest levels of *CTLA4*, *TNFRSF18* (encoding GITR), and the antiinflammatory cytokine *IL10*. Cluster 1 also had the highest frequencies of cells expressing the Th1-related genes *TBX21*, *IL12RB2*, and *CXCR3*, coupled with the lowest levels of *CCR6* and *KLRB1* (encoding CD161) observed in any Treg cluster. Detection of *IFNG* remained rare across the data set, likely because cells were not stimulated prior to scRNA-Seq. These observations led us to define cluster 1 as “Th1-like Tregs” and confirmed that Tregs expressing Th1-related genes maintained a stable Treg transcriptomic signature. Interestingly, cells in this cluster also expressed genes found in T follicular regulatory cells related to T cell–B cell interactions, including *ICOS*, *PRDM1*, and *MAF*, and most markedly, *BATF* (36, 37).

Clusters 2 (classical Tregs) and 3 (activated/HLA-DR^hi^ Tregs) were also dominated by cells expressing Treg-related genes at high levels. Cluster 3 stood out for its striking levels of *HLA-DR* transcripts. Compared with other Treg clusters, cluster 5 (IFN-induced Tregs), and even more so, cluster 4 (CD25^int^CD161^+^ Tregs), expressed Treg-related genes at lower levels and included cells harboring a Teff hash, suggesting some cells in these clusters may be peripherally induced Tregs. Cluster 5 was further characterized by upregulation of IFN-induced genes, mostly triggered by type I IFN signaling (*STAT1*, *STAT2*, *IRF9*) and viral sensing (*IRF1*, *IRF3*, *IRF7*, *ISG15*) or both (38).

### Teff clusters.

scRNA-Seq revealed heterogeneity in oligo JIA SF Teffs, with 7 transcriptionally distinct clusters (Figures 5 and 6). Th1-related genes (*TBX21*, *CXCR3*, *STAT1*) prevailed in Teff clusters. *RORC* detection was limited overall and largely restricted to cluster 10 (Th17); IL-17 was detected in fewer than 30 cells. Of the 758 Teffs in which *TBX21* transcripts were detected, *RORC* was found in 3.6%, indicating few cells with features of nonclassical Th1 cells.

Although both patients had cells in all Teff clusters, the abundance of different subpopulations varied between samples (Figure 5E). Most Teffs from JIA1 (51.6%) had a classical Th1 (cluster 9) or Th17 (cluster 10) profile. By contrast, 47.8% of Teffs from JIA2, who was ANA positive, fell into clusters 6 and 7, characterized by high levels of *IFNG* along with *PDCD1*, *CXCL13*, *BATF*, and *MAF*, markers of the T peripheral helper (Tph) lineage (36). Markers of T cell activation and exhaustion (*HLA-DRB1*, *PDCD1*, *LAG3*, *HAVCR2*) were further upregulated in cluster 6, thus delineated as “activated or HLA-DR^hi^ Tph-like cells.” Other Teff clusters consisted of central memory T cells expressing moderate (cluster 12) to high (cluster 11) levels of CXCR3, accounting for 20%–25% of each patient’s SF Teffs. CTLs, expressing granzymes (*GZMA*, *GZMB*, *GZMK*), perforins (*PRF1*), and cytotoxic granules (*NKG7*) (cluster 8), accounted for the remaining 3%–5% of Teffs.

### TCR repertoire.

**Figure 7:**
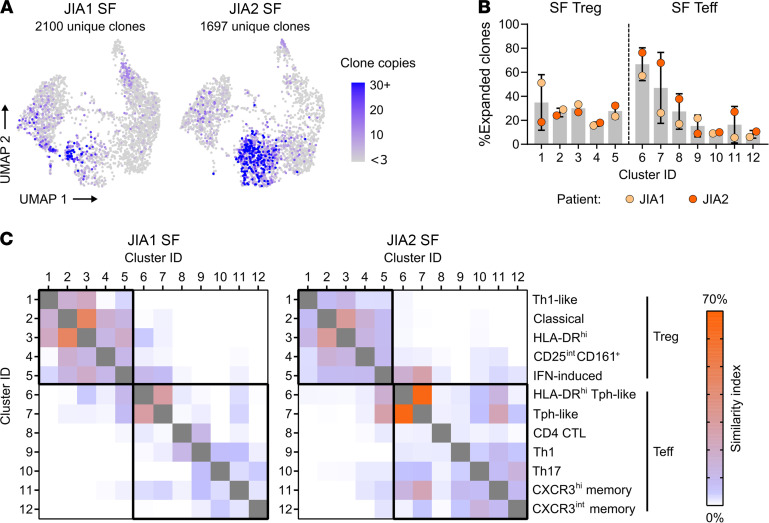
Expanded SF CD4^+^ T cell clones primarily concentrate in clusters of Tph-like cells. Sorted Tregs (CD4^+^CD25^+^CD127^lo^) and Teffs (CD4^+^CD25^–^) from the SF of 2 patients with oligo JIA were evaluated at the single-cell level for T cell receptor (TCR) repertoire analysis with 10x Genomics. (**A**) UMAP projection split by patient, highlighting clones with 3 copies or more across the data set (expanded). A clone is defined by its paired TCRα and TCRβ chain; cells with missing sequencing data for the α and/or β chain were excluded. (**B**) Percentage (mean ± SD) of expanded clones (≥3 copies) per cluster and study subject. (**C**) Similarity in clonotypic composition across clusters for each patient measured with the Morisita-Horn index expressed as a percentage. Clusters are defined as follows: 1) Th1-like Treg, 2) classical Treg, 3) activated/HLA-DR^hi^ Treg, 4) CD25^int^CD161^+^ Treg, 5) IFN-induced Treg, 6) activated/HLA-DR^hi^ Tph-like, 7) Tph-like, 8) CD4^+^ CTLs, 9) Th1 effector memory, 10) Th17 effector memory, 11) CXCR3^hi^ central memory, 12) CXCR3^lo^ central memory. JIA, juvenile idiopathic arthritis; SF, synovial fluid; Tph, T peripheral helper T lymphocytes; CTLs, cytotoxic T lymphocytes; UMAP, uniform manifold approximation and projection.

Complete TCR data (paired CDR3α and CDR3β sequences) were recovered for a total of 5509 cells (89% of the single-cell transcriptomic data set). Clonal expansion was defined as any TCR found in at least 3 sequenced cells; 263 unique clonotypes were expanded (29% of hashed Tregs and 30% of hashed Teffs). Highly expanded Teffs concentrated in Tph clusters, and expanded Tregs were identified in multiple Treg clusters (Figure 7, A and B). Clonotypic composition analysis suggested limited similarity between Th1 (cluster 9) and Th17 (cluster 10) cells (similarity index [SI] = 4% to 6%, Figure 7C). However, the 2 Tph clusters were similarly clonal and dominated by the same clonotypes (SI = 28% to 65%), and so were HLA-DR^hi^ and classical Tregs (SI = 31%–45%). Th1-like Tregs were closely related to other Tregs but not to Teffs, supporting their likely natural Treg origin and their stability. By contrast, the repertoire of IFN-induced Tregs was more closely related to the TCRs found in Teffs, especially Tph-like cells.

## Discussion

Our studies in oligo JIA afford the opportunity to better understand autoimmunity in children by evaluating the interplay between regulatory and effector T cells at the site of active inflammation. In the oligo JIA joint, multiple T cell lineages expressed markers associated with Th1 cells; however, the CD4^+^ T cell subset demonstrated the most profound Th1 polarization. This Th1 signature in CD4^+^ T cells was durable and identified at disease onset and recurrently with arthritic flares. Enrichment of IFN-γ–producing CD4^+^ T cells in the SF was associated with an extended disease course, suggesting this population may drive severe disease. SF Tregs mirrored the Th1 phenotype of proinflammatory effector T cells in the joint but retained their regulatory identity.

Previously, increased IL-17^+^CD4^+^ T cells were reported in JIA SF (7, 8, 39, 40). However, our studies focused on oligo JIA found that only a minority of T cells in oligo JIA SF exhibited Th17 features. This discrepancy likely reflects patient stratification and further demonstrates that JIA encompasses different forms of chronic inflammatory arthritis that are characterized by distinct biology. Our results are concordant with prior work limited to oligo and the closely related seronegative polyarticular JIA, which also highlighted a predominance of cells expressing either CXCR3 or IFN-γ in JIA SF (9–12). Expanding on these findings, we demonstrated a robust Th1 gene expression signature in CD4^+^ T cells found in oligo JIA SF, confirmed persistent Th1 enrichment in the joint longitudinally, and documented an association between the frequency of IFN-γ^+^CD4^+^ T cells in the SF and severe disease.

Our studies provide further clarity on the origin of IFN-γ–producing CD4^+^ T cells in oligo JIA SF and whether classical Th1 (derived from naive CD4 T cells) or nonclassical Th1 cells (derived from Th17 cells) predominate in the joints of children with oligo JIA (13–16, 25). Both subsets express the traditional Th1 markers (T-bet, CXCR3, IFN-γ), but nonclassical Th1 cells also retain expression of some Th17 markers (RORγt, CCR6, CD161, IL-17) (16, 17, 41). In this study, we found little evidence for coexpression of Th1 and Th17 markers in single cells. Similar to prior findings, IFN-γ^+^IL-17^+^ cells are rare in the joints of oligo JIA patients, with percentages typically less than 3% of total SF CD4^+^ Tmem cells (12–14). Further, less than 4% of T-bet–expressing Teffs in scRNA-Seq coexpressed RORγt, and the repertoire of Th1 and Th17 clusters were not highly related. While we cannot exclude a pathological role for the mixed Th1 and Th17 population, our results indicate that nonclassical Th1 cells represent a small fraction of IFN-γ–producing cells in oligo JIA SF.

CD161 is a C-type lectin-like receptor that is found on Th17 cells (25). Increased frequencies of IFN-γ^+^CD161^+^CD4^+^ T cells in oligo JIA SF, found in this study and others, have often been interpreted as an indicator of Th17 origin and a marker of nonclassical Th1 cells (13, 14). This interpretation relies on the assumption that CD161 solely indicates Th17 lineage (13–16, 25). However, CD161 is expressed on many other T cell types (42). Fergusson et al. proposed that CD161 also defines a population of innate-like T cells poised to produce IFN-γ in response to IL-12 and IL-18 (42). In our bulk transcriptomic studies, increased expression of IL-12 and IL-18 receptor was found in SF CD4^+^ T cells. By scRNA-Seq, *KLRB1* (encoding CD161) was expressed in virtually all effector memory clusters but was remarkably absent in central memory cells. Both observations support the model put forth by Fergusson et al. and suggest that CD161 is a costimulatory molecule found on CD4 T cells capable of producing IFN-γ.

SF Tregs in oligo JIA adopt a Th1 phenotype with close to 80% on average expressing CXCR3 and increased transcription of *TBX21* (encoding T-bet). A small but significant fraction of SF Tregs also produce IFN-γ. Th1-like Tregs have been described previously, although not in JIA (43–49). Th1 polarization allows Tregs to accumulate at sites of Th1 inflammation, which is beneficial in circumstances such as infection (46, 48). However, in some inflammatory environments, Th1-like Tregs can convert into effectors that contribute to inflammation (43, 47, 49). In oligo JIA SF, CXCR3^+^ Tregs maintained their suppressive function and lineage-defining methylation patterns, suggesting preserved Treg identity. These results differ from prior studies that found Teffs from the SF of JIA patients to be resistant to Treg-mediated suppression (21, 22). These discrepant results may be secondary to the population of Tregs cocultured with SF Teffs in suppression assays. Haufe et al. defined Tregs as CD4^+^CD25^+^ T cells, and this population may actually contain activated, nonregulatory T cells (21, 50). While Wehrens and colleagues evaluated the bulk SF Treg population (CD4^+^CD25^+^CD127^lo^ T cells), our studies focused on CXCR3^+^ Tregs (22). The data presented herein, as well as the work of others, suggests that CXCR3^+^ Tregs in certain environments may have enhanced suppressive function compared with other Treg subsets (22, 46). We cannot rule out that the small proportion of IFN-γ^+^ SF Tregs detected by flow cytometry represents an unstable Treg population. Without prior stimulation, *IFNG* transcripts in our scRNA-Seq studies remained rare, and the Treg signature of IFN-γ–producing cells could not be determined. Yet, the Treg signature was preserved in SF Tregs coexpressing other Th1-related genes, suggesting Treg stability in the arthritic joint.

Single-cell immunophenotyping methods have increasingly uncovered the diversity of human Tregs (33, 45, 51). Even in the Th1-polarized environment of oligo JIA SF, we identified several Treg subpopulations, including Th1-like, IFN-induced, HLA-DR^+^, and CD161^+^ Tregs. HLA-DR^+^ and CD161^+^ Tregs have been identified previously in JIA and have been shown by others to maintain suppressive capacity, even in inflammatory environments (52–55). Herein, we showed that Th1-like Tregs maintained robust expression of Treg-related genes, indicating that this Treg subset also maintains regulatory function in oligo JIA. The population of Tregs displaying a transcriptional fingerprint indicative of viral sensing and IFN signaling is less well defined in the literature, although very similar transcriptomic signatures have been observed in tumor-infiltrating CD4^+^ T cells (56). Further work is needed to understand the functionality of IFN-induced Tregs in oligo JIA.

Single-cell profiling also identified a Teff population with features reminiscent of Tph cells, which were originally described in rheumatoid arthritis synovium and promote B cell responses (36). Interestingly, this Tph-like population was found at higher frequency and clonality in the ANA-positive patient. This aligns with the recent observation of an increased frequency of CD4^+^ T cells that produce the Tph-related cytokine IL-21 in ANA-positive JIA patients (12). Yet, the ability of Tph-like cells in JIA SF to promote B cell maturation and pathological autoantibody production remains unknown. In addition, a cluster of CD4^+^ T cells expressing multiple markers of cytotoxicity, which are most commonly associated with NK and CD8^+^ T cells, was uncovered. While still debated, it has been proposed that CD4^+^ CTLs kill cells in an MHC class II–restricted manner (57). CD4^+^ CTLs have been most commonly identified in the context of viral infection or antitumor immunity and appear to be closely related to Th1 cells (58, 59). Interestingly, scRNA-Seq studies in patients with COVID-19 identified a similar population of CD4^+^ CTLs alongside cytotoxic Tfh cells; and the abundance of these cytotoxic populations correlated with limited Treg responses and severe disease (60, 61). The identification of CD4^+^ CTLs in oligo JIA suggests that this population of CD4^+^ T cells may be important in Th1-mediated autoimmunity, particularly in inflamed peripheral tissues.

Our human-based research has limitations, which we attempted to address by evaluating a well-defined study group (oligo JIA) and accounting for disease duration and treatment. Although every effort was made to focus on oligo JIA, not all patients underwent testing for HLA-B27 and it is possible that some children with enthesitis-related arthritis were included in this study. The young age of onset and female predominance in this cohort is a characteristic of oligo JIA and suggests that most patients were classified correctly. These studies were performed with T cells obtained from the SF, which may not fully represent the T cell populations in synovial tissue. Synovial biopsies are not routinely performed in children with oligo JIA, and our access to such tissue samples was limited. Similarly, our studies of Treg function were restricted to in vitro suppression assays that measure Teff proliferation and cytokine production and may not reflect the complex interactions that occur in vivo between Tregs and other non–T cell populations. In addition, only 2 patients were evaluated by scRNA-Seq, but these results were interpreted alongside data derived from a larger patient cohort.

Our study provides important insights into the CD4^+^ T cells that contribute to Th1 responses in oligo JIA. Our results suggest that controlling Th1-mediated inflammation, potentially via Treg-based approaches delivered to the joint, may be an important strategy to restore tolerance in oligo JIA.

## Methods

### Patients and samples.

A board-certified pediatric rheumatologist reviewed the medical records to gather clinical data and confirm the diagnosis. Patients within 6 months of symptom onset were classified as new-onset oligo JIA. ANA positivity was defined by a positive titer at any level on a single occasion. PB and/or discarded SF samples were collected from patients with oligo JIA defined by the ILAR criteria (1). As outlined in the ILAR criteria, children with enthesitis, known HLA-B27 positivity, psoriasis, and/or family history of psoriasis in a first-degree relative at the time of enrollment and sample collection were excluded. When available, medical records were reviewed prior to publication of this study to identify interval changes in the clinical history of enrolled study subjects and determine whether the JIA classification had changed. PB samples were also obtained from controls. Clinical characteristics of the study cohort are available in Table 1 and Supplemental Table 1.

### Cell isolation.

Mononuclear cells were isolated from the PB and SF by Ficoll density gradient centrifugation (GE Healthcare, now Cytiva), cryopreserved in liquid nitrogen, and subsequently thawed into RPMI (HyClone) with 10% FBS (MilliporeSigma), penicillin 100 IU/mL (Corning Cellgro), streptomycin 100 μg/mL (Corning Cellgro), nonessential amino acids (Corning Cellgro), sodium pyruvate 1 mM (Corning Cellgro), and HEPES 10 mM (Thermo Fisher Scientific). After thawing, PBMCs and synovial fluid mononuclear cells (SFMCs) were incubated overnight before downstream experiments.

### Flow cytometry.

PBMCs and SFMCs were left unstimulated or stimulated with a cocktail of PMA, ionomycin, and brefeldin A (BD Biosciences) for 4 hours. Cells were stained for viability and surface markers, then fixed and permeabilized (eBioscience) before intracellular staining. Antibodies and dyes are listed in Supplemental Table 2. Data were acquired on an LSRFortessa (BD Biosciences) and analyzed using FlowJo.

### Bulk RNA-Seq.

After enrichment for CD4^+^ T cells (Miltenyi Biotec), Tregs (CD3^+^CD4^+^CD25^+^CD127^lo^) and Teffs (CD3^+^CD4^+^CD25^–^) were isolated by FACS. RNA was extracted from 10,000 cells of each population (QIAGEN) and sequenced using the Smart-seq2 platform at Broad Technology Labs (62). Sequencing was run in 2 batches on an Illumina NextSeq500 sequencer, with both runs relying on identical pipelines.

Samples were analyzed and quality-controlled as previously described (28). Briefly, the bcbio-nextgen RNA-Seq analysis pipeline was used (https://bcbio-nextgen.readthedocs.org/en/latest/). Trimmed reads that passed quality control with FastQC (http://www.bioinformatics.babraham.ac.uk/projects/fastqc/) were aligned to UCSC build GRCh37 of the human genome (*Homo sapiens*), augmented with transcript information from Ensembl release 90 using STAR (63). Counts of reads aligning to known genes were computed using featureCounts, and transcripts per million (TPM) measurements per isoform were determined through quasi-alignment using Salmon (64, 65). Counts per gene estimated by tximport from Salmon quasi-alignments were used for downstream analyses in R version 3.6.2 (https://www.R-project.org/).

To determine the impact of running samples in 2 batches, PCA was performed on full gene expression profiles after alignment and scrutinized for association with various covariates, including subject characteristics and technical factors. The PCA uncovered a minor technical batch effect, which was addressed by regressing out the sequencing run variable when modeling the data and estimating log_2_ fold changes in differential gene expression analysis. Two patients whose diagnosis evolved from oligoarticular to enthesitis-related arthritis or psoriatic JIA contributed paired samples to this transcriptomic study. In PCA, samples from these patients did not cluster apart from other JIA samples obtained from the same compartment (PB or SF); therefore, to maximize the statistical power of our analysis, the patients with enthesitis-related arthritis and psoriatic JIA were not excluded. Similarly, patient and control PB samples did not cluster apart in PCA; therefore, transcriptomic analyses of PB T cell populations jointly considered JIA and control subjects.

Differential gene expression analysis was run using DESeq2 version 1.22.2 (66). DESeq2-estimated log_2_ fold changes in gene expression were corrected using the normal method implemented in the lfcShrink function (66). Rank-corrected log_2_ fold change values served as input for a cutoff-free GSEA using clusterProfiler version 3.14.3 (67). GSEA compared sequencing data with the Reactome database (immune system and metabolism subsets), the human Treg gene set, and the mouse peripheral/induced Treg gene set (29, 30, 68).

### Treg suppression assays and CpG methylation studies.

For these analyses, Teffs (CD3^+^CD4^+^CD25^–^) as well as bulk or CXCR3^+^ and CXCR3^–^ Tregs (CD3^+^CD4^+^CD25^+^CD127^lo^) were isolated using FACS. The suppressive capacity of SF Tregs was assessed against autologous SF Teffs as well as against PB Teffs from third-party controls; the suppressive capacity of control PB Tregs was assessed against autologous PB Teffs. Teffs were labeled with CellTrace Violet (Invitrogen), stimulated with anti-CD2/CD3/CD28 beads (Miltenyi Biotec), and cocultured with Tregs at various ratios. After 4 days, Teff cell division was assessed by dye dilution using flow cytometry (LSRFortessa, BD Biosciences), and cytokine concentrations in culture supernatants were measured by Luminex FlexMap 3D assay system per the manufacturer’s instructions. For methylation analysis, cells were pelleted and snap-frozen. EpigenDx extracted DNA and performed the bisulfite conversion pyrosequencing for methylation analysis of the cytosine guanine dinucleotide sites listed in Supplemental Table 3.

### scRNA-Seq with TCR repertoire.

First, 5000 Tregs and 5000 Teffs were FACS-sorted from the SF of 2 patients as for bulk RNA-Seq, stained with hashing antibodies, and pooled together for processing in a single batch (69). After encapsulation and barcoding (10x Genomics), cells were lysed, and cDNA was prepared to create a 5′ gene expression library; a variable, diversity, and joining gene-enriched library for TCR repertoire analysis; and a hashtag oligonucleotide library for source sample identification (70). Libraries were sequenced using Illumina NovaSeq 6000. Demultiplexing and gene alignment were performed with CellRanger (10x Genomics) (70).

After gene alignment, the complete matrix of raw features generated by CellRanger was imported in R version 3.6.2 using the Read10X function of Seurat version 3.1.5 (71). Droplets with fewer than 500 genes, fewer than 800 unique molecular identifiers (UMIs), a log_10_(gene) to log_10_(UMI) ratio less than 0.85, or a mitochondrial to genomic transcript ratio more than than 0.15 were excluded, leaving a total of 7304 cells. Genes detected in 10 cells or less were filtered out, leaving a total of 13,772 genes. Hashing data were normalized using centered log ratios, then demultiplexed using Seurat’s HTODemux function with the default “clara” method and a positive quantile cutoff set to 0.98. Cells with unknown or multiple hash detection were excluded at this stage, leaving a total of 6190 cells for analysis.

The transcriptomic data that passed all quality control steps above were normalized with Seurat’s SCTransform function, using patient ID as a variance stabilization covariate to limit clustering by patient and searching for the top 3200 most variable features in the data set. To enable clustering by transcriptomic profile regardless of clonal identity, the list of most variable features was manually filtered out for *TRAV*, *TRAJ*, *TRBV*, and *TRBJ* genes, then reduced to a length of 3000 features.

PCA was run on the resulting filtered list of top 3000 most variable features. Beyond the 11th principal component (PC), each new PC contributed little more to explaining the overall variation in the data set. We therefore used 11 PCs for Louvain clustering analysis with Seurat’s FindNeighbors and FindClusters functions and settled on a resolution of 1.0 to define the clusters in the data set. All visualizations of the single-cell data were based on a uniform manifold approximation and projection (UMAP) dimensionality reduction, computed with uwot version 0.1.5 and using 22 PCs.

For cluster characterization, we used Seurat’s FindMarker function alongside a manual search for genes of interest. Unless results from the marker search highlighted another profile (notably, high expression of genes suggestive of a specific metabolic or mitotic cell state), we sought to categorize clusters as presenting a predominately Treg or Teff transcriptomic profile. We thus defined clusters with simultaneous expression of *FOXP3*, *IL2RA*, *IKZF2*, *CTLA4*, and *TNFRSF18* at moderate to high levels as “Treg clusters.” In contrast, clusters with low to no detection of these key Treg genes were defined as “Teff clusters.” The Treg versus Teff identity assignment was further informed by the relative proportion of cells hashed with a Treg or Teff antibody within the corresponding cluster, although a small fraction of cells with a Treg hash clustered with cells presenting a Teff transcriptomic profile and vice versa. For further characterization of Treg and Teff clusters, we qualitatively contrasted their gene expression patterns with known markers of different T cell states (including Th1, Th17, T follicular helper cells, and activated/circulating lymphocytes).

For the TCR repertoire component, we corrected the raw copy number of each clonotype identified by CellRanger vdj to account solely for the 6190 cells retained in prior steps of the analysis. TCR repertoire characteristics were studied using custom R code and stored in the metadata slot of the Seurat object. The similarity in clonotypic composition between 2 clusters is defined as 1-MH, where MH is the Morisita-Horn dissimilarity index calculated using the vegdist function of the vegan R package version 2.5.7 (72).

### Data accessibility.

Raw bulk and scRNA-Seq as well as TCR repertoire data are available at ImmPort (https://www.immport.org) under study accession SDY1777. An interactive tool for exploration of the analyzed data sets is available at https://amjule.shinyapps.io/oligo-JIA/

### Statistics.

One-way ANOVA with Tukey’s correction for multiple comparisons was used to compare differences across more than 2 study groups for normally distributed data. Paired or unpaired 2-tailed *t* tests were used for analyses restricted to 2 study groups with normally distributed data. Significance was defined as a *P* value less than 0.05. For GSEA, significance was defined at an FDR (*q* value) less than 0.05.

### Study approval.

These studies were approved by the IRB at Boston Children’s Hospital (IRB protocols 07-09-0375 and IRB-P00005723). Written informed consent (and assent when appropriate) was provided by participants and/or legal guardians.

## Author contributions

LAH and AMJ conceived and designed the study. LAH supervised the research. PAN and TC contributed to the experimental design. AMJ, KJH, MT, JN, JAL, and LAH performed the experiments and acquired the data. KW and MGA assisted in the scRNA-Seq experiments and analysis. KJH, MT, SMC, MHC, EMC, FD, MMH, JH, OH, EJ, JL, MSL, EM, JER, MBFS, RPS, PYL, PAN, and LAH recruited patients and collected samples. AMJ, KJH, JN, and LAH analyzed the data. AMJ and LAH drafted the manuscript with input from PAN and PYL. All authors edited the manuscript and approved the final version.

## Supplementary Material

Supplemental data

Supplemental data set 1

## Figures and Tables

**Table 1:**
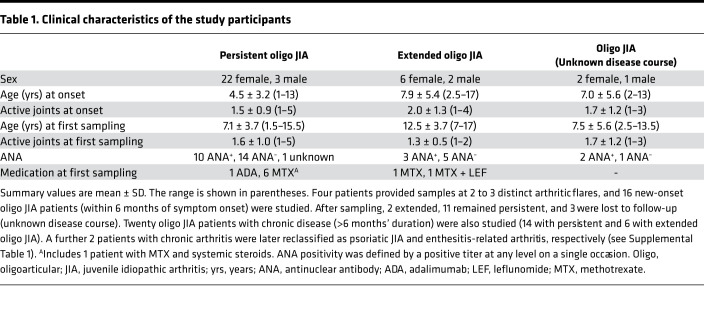
Clinical characteristics of the study participants

## Version 1 08/17/2021

In-Press Preview

## Version 2 09/22/2021

Electronic publication
