# Supplementary material for: Th1 polarization defines the synovial fluid T cell compartment in oligoarticular juvenile idiopathic arthritis
Source: JCI Insight. 2021 Sep 22;6(18):e149185. doi: 10.1172/jci.insight.149185 (PMC8492302; doi:10.1172/jci.insight.149185)
Supplement: Supplemental data [file jciinsight-6-149185-s215.pdf]

SUPPLEMENTARY FIGURES

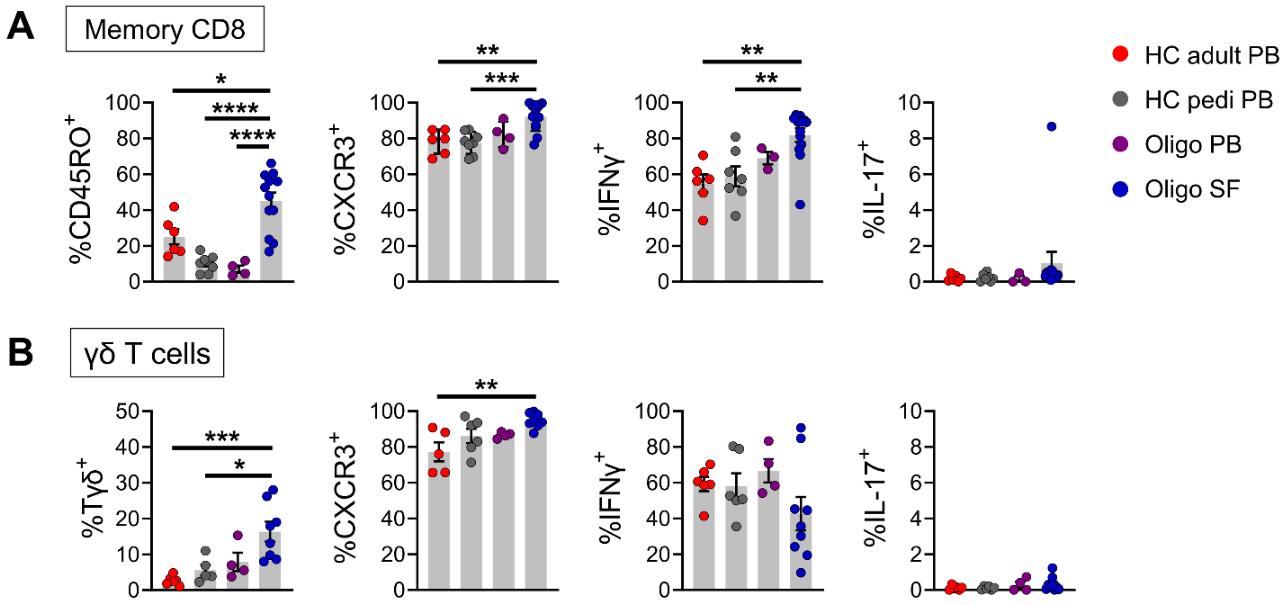

Figure S1: CD8 memory T cells and γδ T cells are enriched in the SF of oligo JIA patients, and upregulate Th1 markers. A) Percent of total $CD8^+$ T memory (Tmem, $CD3^+CD8^+CD45RO^+$) cells among total $CD8^+$ T cells and of $CD8^+$ Tmem expressing different cytokines and chemokines in the SF of oligo JIA patients (n=12-13) and in the PB of patients (n=3-4) and controls (adult: n=6, pediatric: n=7-8). B) Percent of total γδ T lymphocytes ($CD3^+T\gamma\delta^+$) and of γδ T cells expressing different cytokines and chemokines in the SF of oligo JIA patients (n=8-9) and in the PB of patients (n=4) and controls (adult: n=5-6, pediatric: n=5-6). HC, healthy control; pedi, pediatric; oligo, oligoarticular juvenile idiopathic arthritis; PB, peripheral blood; SF, synovial fluid. Summary data on all bar graphs are mean ± standard error. P-value <0.05 (*); <0.01 (**), <0.001 (***), <0.0001 (****). Statistical testing: one-way ANOVA followed by multiple t-tests with Turkey correction.

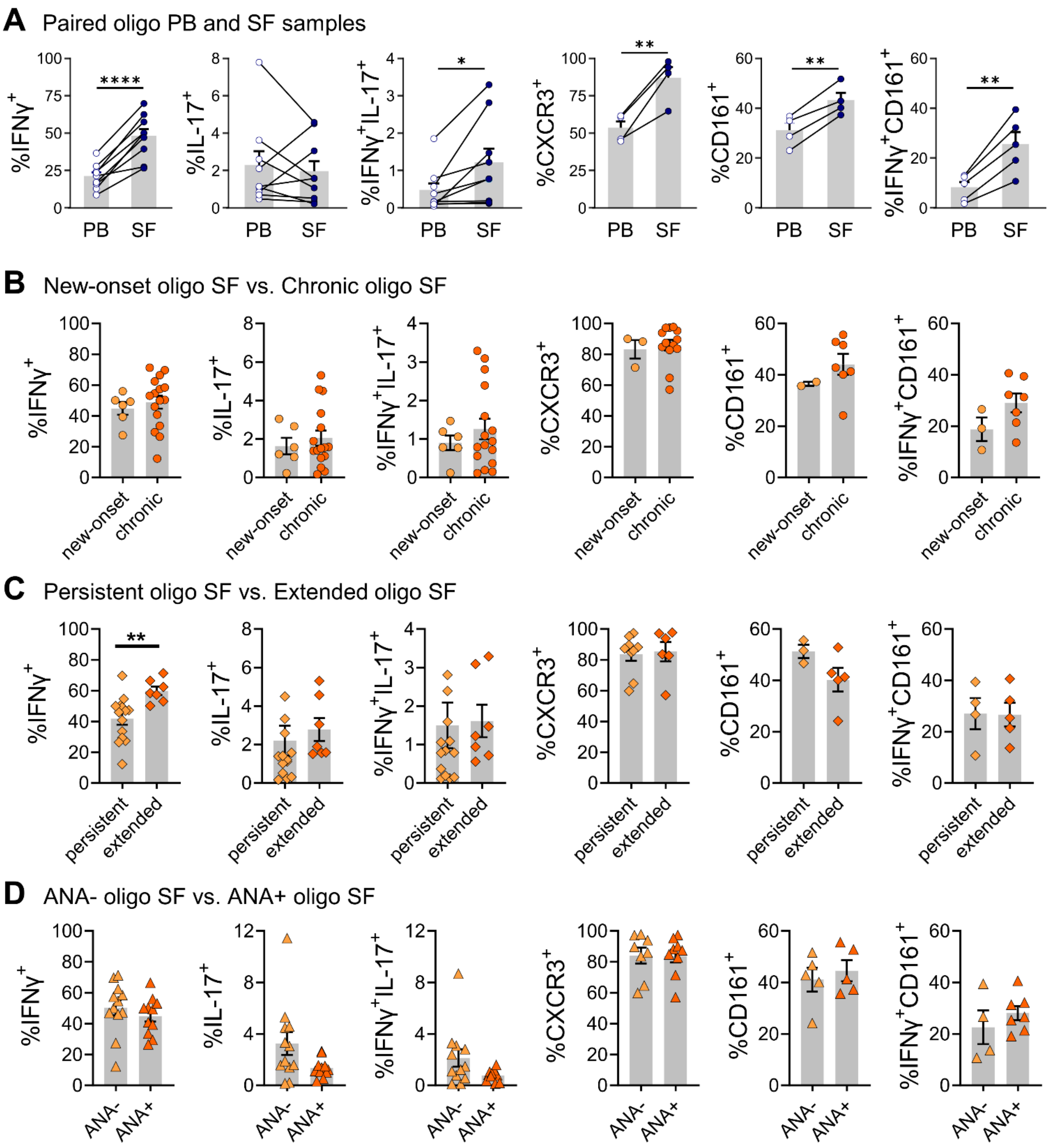

Figure S2: Th1 polarization of $CD4^+$ Tmem cells in the SF of new-onset and chronic oligo JIA patients. A) Percent of $CD4^+$ Tmem ($CD3^+CD4^+CD45RO^+$) cells expressing different cytokines and chemokines in paired PB and SF samples from oligo JIA patients (n=4-9). B) Percent of $CD4^+$ Tmem cells expressing different cytokines and chemokines in oligo JIA SF samples obtained at disease onset (n=2-5) or in chronic disease phase (n=7-16). C) Percent of $CD4^+$ Tmem cells expressing different cytokines and chemokines in the SF of patients progressing to persistent (n=3-13) or extended (n=5-7) forms of oligo JIA. D) Percent of $CD4^+$ Tmem cells expressing different cytokines and chemokines in the SF of oligo JIA patients with (n=5-11) or without (n=4-12) antinuclear autoantibody (ANA). All analyses were gated on $CD3^+CD4^+CD45RO^+$ cells ($CD4^+$ Tmem). PB, peripheral blood; SF, synovial fluid; oligo, oligoarticular juvenile idiopathic arthritis; ANA, antinuclear autoantibody. Summary data on all bar graphs are mean ± standard error. P-value <0.05 (*); <0.01 (**), <0.001 (***), <0.0001 (****). Statistical testing: Paired (a) and unpaired (b and c) t-tests.

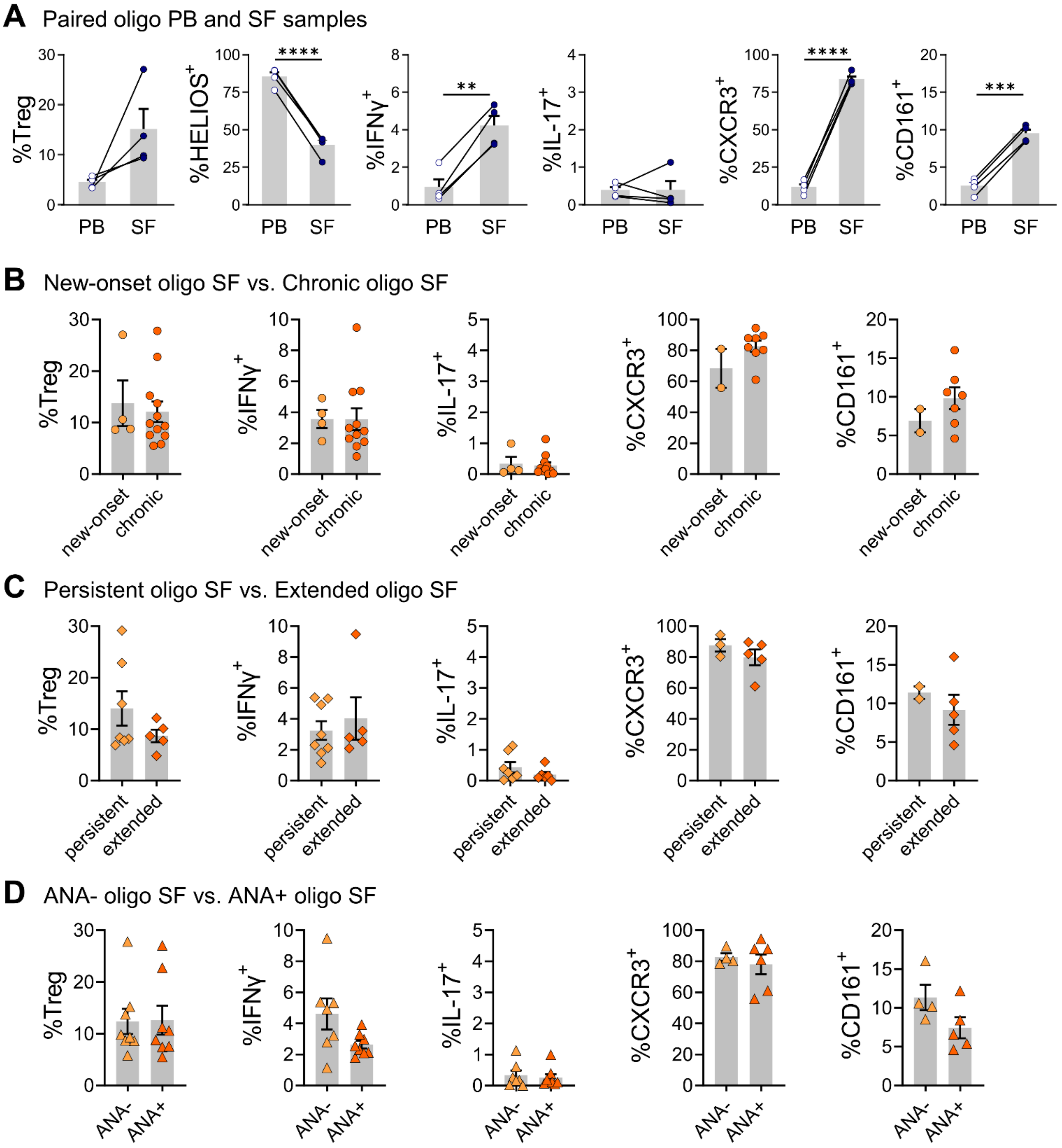

Figure S3: Th1 polarization of regulatory T (Treg) cells in the SF of new-onset and chronic oligo JIA patients. A) Percent of total Treg ($CD25^+CD127^{lo}FOPX3^+$) among $CD4^+$ T cells, and of Treg cells expressing different cytokines and chemokines in paired PB and SF samples from oligo JIA patients (n=4). B) Percent of total Treg ($CD25^+CD127^{lo}FOPX3^+$) among $CD4^+$ T cells, and of Treg cells expressing different cytokines and chemokines in oligo JIA SF samples obtained at disease onset (n=2-4) or in chronic disease phase (n=7-12). C) Percent of total Treg ($CD25^+CD127^{lo}FOPX3^+$) among $CD4^+$ T cells and of Treg cells expressing different cytokines and chemokines in the SF of patients progressing to persistent (n=2-8) or extended (n=5) forms of oligo JIA. D) Percent of total Treg ($CD25^+CD127^{lo}FOPX3^+$) among $CD4^+$ T cells and of Treg cells expressing different cytokines and chemokines in the SF of oligo JIA patients with (n=5-8) or without (n=4-8) antinuclear autoantibody (ANA). PB, peripheral blood; SF, synovial fluid; oligo, oligoarticular juvenile idiopathic arthritis; ANA, antinuclear autoantibody. Summary data on all bar graphs are mean ± standard error. P-value <0.05 (*); <0.01 (**), <0.001 (***), <0.0001 (****). Statistical testing: Paired (a) and unpaired (b and c) t-tests.

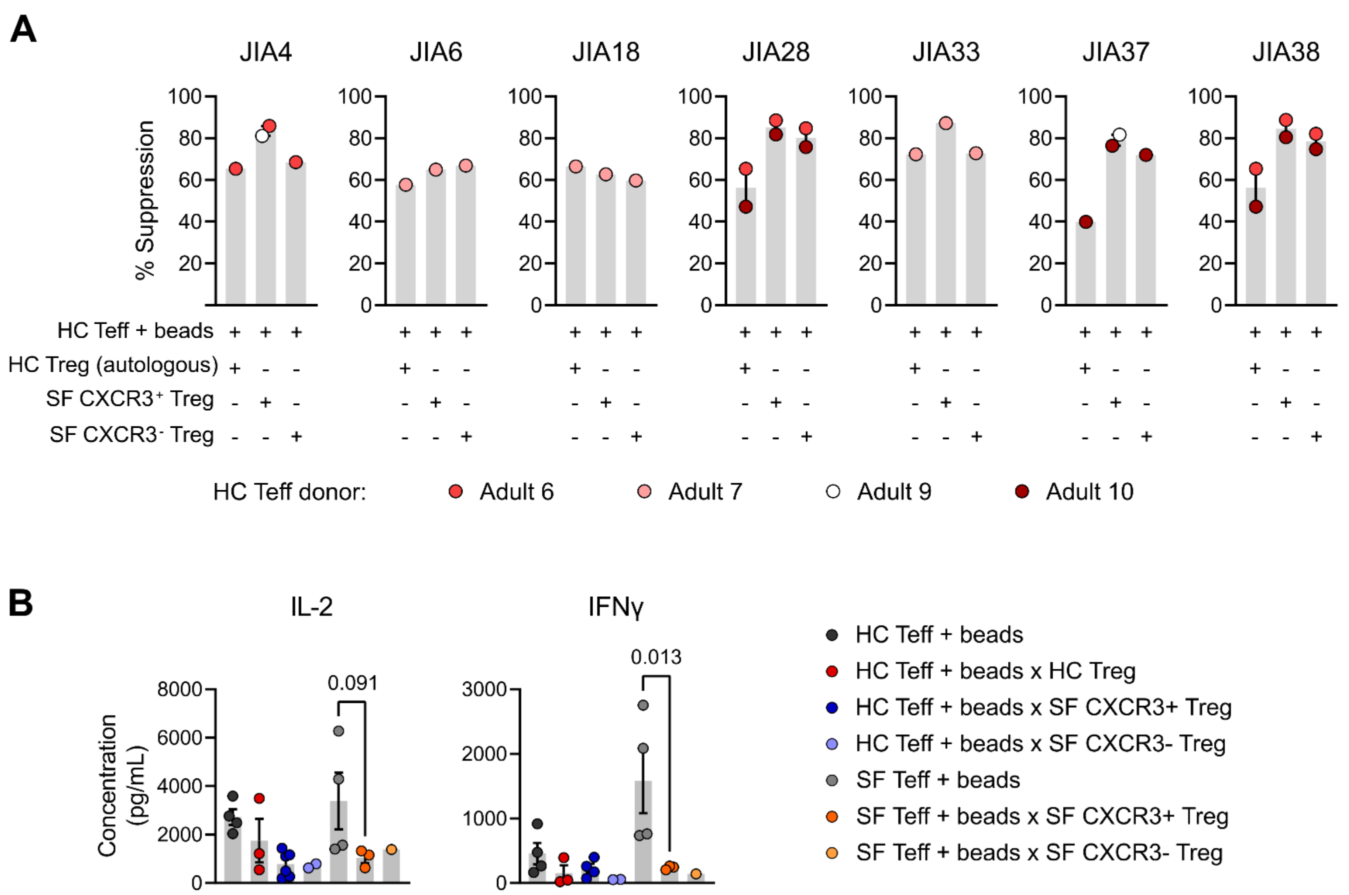

Figure S4: Th1-like Tregs maintain the ability to suppress Teff proliferation and cytokine production. A) Per patient percent suppression (mean ± standard error) in the proliferation of Teff cells ($CD4^+CD25^-$) from a third-party control after co-culture at a ratio of 1:1 with autologous PB Tregs ($CD4^+CD25^+CD127^{lo}$), oligo JIA $CXCR3^+$ SF Tregs or oligo JIA $CXCR3^-$ SF Tregs. Given the small number of subjects in each experiment, statistical testing was not preformed. B) Cytokine concentration (pg/mL, mean ± standard error) detected in the supernatant of suppression assay co-cultures. Teffs from healthy controls were stimulated with anti-CD2/CD3/CD28 beads alone or co-cultured at a ratio of 1:1 with autologous Tregs, oligo JIA $CXCR3^+$ SF Tregs, or oligo JIA $CXCR3^-$ SF Tregs. Similarly, oligo JIA SF Teffs were stimulated with beads and co-cultured at a ratio of 1:1 with autologous oligo JIA $CXCR3^+$ SF Tregs or oligo JIA $CXCR3^-$ SF Tregs. The depicted data represents 4 independent experiments conducted with 4 healthy controls and 5 oligo JIA patients. HC, healthy control; oligo, oligoarticular; JIA, juvenile idiopathic arthritis; PB, peripheral blood; SF, synovial fluid. Statistical testing: one-way ANOVA followed by selected t-tests with Turkey correction.

SUPPLEMENTARY TABLES

Table S1: Clinical Characteristics of the Study Participants and Samples Contributed to the Studies.

Subject	Sample	Group	JIA subtype at sampling	JIA subtype end of study	Sex	Age (yrs) at onset	Age (yrs) at sampling	Joint involvement at onset	Joint involvement at sampling	ANA	Uveitis	Treatment at sampling	Flow	RNA-seq	Other
Adult 1	PB	Control	NA	NA	F	NA	24-25	NA	NA	NA	NA	NA	Yes	No	No
Adult 2	PB	Control	NA	NA	F	NA	24-25	NA	NA	NA	NA	NA	Yes	No	No
Adult 3	PB	Control	NA	NA	M	NA	35-36	NA	NA	NA	NA	NA	Yes	Bulk	Suppr assay
Adult 4	PB	Control	NA	NA	M	NA	28	NA	NA	NA	NA	NA	Yes	No	No
Adult 5	PB	Control	NA	NA	M	NA	35	NA	NA	NA	NA	NA	No	Bulk	No
Adult 6	PB	Control	NA	NA	F	NA	26-28	NA	NA	NA	NA	NA	Yes	No	Suppr assay, Methylation
Adult 7	PB	Control	NA	NA	F	NA	35-36	NA	NA	NA	NA	NA	Yes	Bulk	Suppr assay, Methylation
Adult 8	PB	Control	NA	NA	F	NA	28-29	NA	NA	NA	NA	NA	Yes	Bulk	No
Adult 9	PB	Control	NA	NA	M	NA	36	NA	NA	NA	NA	NA	Yes	No	Suppr assay, Methylation
Adult 10	PB	Control	NA	NA	F	NA	22	NA	NA	NA	NA	NA	No	No	Suppr assay
Pedi 1	PB	Control	NA	NA	F	NA	16	NA	NA	NA	NA	NA	Yes	Bulk	No
Pedi 2	PB	Control	NA	NA	F	NA	11.5	NA	NA	NA	NA	NA	Yes	Bulk	No
Pedi 3	PB	Control	NA	NA	F	NA	7	NA	NA	NA	NA	NA	Yes	Bulk	No
Pedi 4	PB	Control	NA	NA	M	NA	11	NA	NA	NA	NA	NA	Yes	Bulk	No
Pedi 5	PB	Control	NA	NA	F	NA	6	NA	NA	NA	NA	NA	Yes	Bulk	No
Pedi 6	PB	Control	NA	NA	M	NA	6.5	NA	NA	NA	NA	NA	Yes	No	No
Pedi 7	PB	Control	NA	NA	M	NA	7	NA	NA	NA	NA	NA	Yes	No	No
Pedi 8	PB	Control	NA	NA	M	NA	14	NA	NA	NA	NA	NA	Yes	No	No
JIA 01	PB, SF	Oligo	Persistent	Persistent	F	4.5	9	2	1	-	No	ADA	Yes	Single-cell (SF)	No
JIA 02	PB, SF	Oligo	New-onset	Persistent	F	2	2	1	1	+	No	None	No	Bulk	No
JIA 02 (repeat)	SF	Oligo	Persistent	Persistent	F	2	4	1	1	+	No	ETA	Yes	Single-cell (SF)	No
JIA 03	SF	Oligo	Persistent	Persistent	F	3.5	9	1	1	-	No	None	Yes	No	No
JIA 03 (repeat)	PB, SF	Oligo	Persistent	Persistent	F	3.5	9.5	1	Unknown	-	No	None	No	Bulk	No
JIA 04	PB, SF	Oligo	New-onset	Persistent	F	10	10	1	1	+	No	None	No	Bulk	No
JIA 04 (repeat)	SF	Oligo	Persistent	Persistent	F	10	13	1	1	+	No	MTX	Yes	No	No
JIA 04 (repeat)	SF	Oligo	Persistent	Persistent	F	10	16	1	1	+	No	MTX	No	No	Suppr assay

JIA 05	PB, SF	Oligo	Persistent	Extended	F	2.5	10	1	1	-	No	None	No	Bulk	No
JIA 05 (repeat)	PB, SF	Oligo	Persistent	Extended	F	2.5	14.5	1	1	-	No	ETA	Yes	No	No
JIA 06	PB, SF	Oligo	New-onset	Persistent	F	13	13	1	1	-	No	None	Yes	Bulk	Suppr assay
JIA 07	PB, SF	Oligo	New-onset	Persistent	F	3.5	3.5	2	2	-	No	None	No	Bulk	No
JIA 08	PB, SF	Oligo	New-onset	Persistent	F	5	5	2	2	-	Unknown	None	Yes	No	No
JIA 09	PB	Oligo	New-onset	Persistent	F	1.8	1.8	1	1	-	No	None	Yes	No	No
JIA 10	PB	Oligo	New-onset	Persistent	F	5	5	1	1	-	No	None	Yes	No	No
JIA 11	PB	Oligo	New-onset	Persistent	M	4	4	5	5	+	No	MTX*	Yes	No	No
JIA 12	PB	Oligo	New-onset	Persistent	M	4	4	1	1	+	No	None	Yes	No	No
JIA 13	SF	Oligo	New-onset	Persistent	F	2.5	2.5	2	4	+	Unknown	None	Yes	No	No
JIA 14	PB	Oligo	New-onset	Persistent	F	2	2.5	1	1	+	Yes	MTX	Yes	No	No
JIA 15	PB, SF	Oligo	Persistent	Persistent	F	2	12	2	1	-	No	MTX	No	Bulk	Methylation
JIA 16	PB, SF	Oligo	Persistent	Persistent	F	3	9	2	2	+	No	MTX	Yes	Bulk	Methylation
JIA 17	PB, SF	Oligo	Persistent	Persistent	F	3.5	10	1	1	-	Yes	MTX	Yes	Bulk	Methylation
JIA 18	SF	Oligo	Persistent	Persistent	F	5.5	6	1	1	+	No	None	Yes	No	Suppr assay
JIA 19	SF	Oligo	Persistent	Persistent	F	1	7	Unknown	2	+	No	None	Yes	No	No
JIA 20	SF	Oligo	Persistent	Persistent	F	4	10	1	1	-	Yes	None	Yes	No	No
JIA 21	SF	Oligo	Persistent	Persistent	F	2	4.5	2	2	-	Yes	None	Yes	No	Methylation
JIA 22	SF	Oligo	Persistent	Persistent	M	5	6	1	1	-	Unknown	None	Yes	No	No
JIA 23	SF	Oligo	Persistent	Persistent	F	13	15.5	1	1	+	No	None	Yes	No	No
JIA 24	SF	Oligo	Persistent	Persistent	F	6	11.5	1	2	-	No	None	Yes	No	No
JIA 25	PB, SF	Oligo	New-onset	Extended	M	15.5	16	2	2	+	No	None	No	Bulk	No
JIA 26	PB, SF	Oligo	New-onset	Extended	F	17	17	1	2	-	No	None	Yes	Bulk	No
JIA 27	PB, SF	Oligo	Persistent	Extended	F	6	8.5	1	1	-	No	None	Yes	No	No
JIA 28	SF	Oligo	Extended	Extended	F	6	14.5	1	1	-	No	None	Yes	No	Suppr assay
JIA 29	SF	Oligo	Extended	Extended	F	4	15	4	1	+	Yes	None	Yes	No	No
JIA 30	SF	Oligo	Extended	Extended	M	4	7	2	1	-	No	LEF, MTX	Yes	No	No
JIA 31	SF	Oligo	Extended	Extended	F	8.5	12	4	Unknown	+	No	MTX	Yes	No	No
JIA 32	PB, SF	Oligo	New-onset	Unknown	F	6	6.5	1	1	-	No	None	No	Bulk	No
JIA 33	SF	Oligo	New-onset	Unknown	M	2	2.5	3	3	+	No	None	Yes	No	Suppr assay Methylation
JIA 34	SF	Oligo	New-onset	Unknown	F	13	13.5	1	1	+	Unknown	None	Yes	No	No
JIA 35	PB, SF	Psoriatic	Persistent	Psoriatic	M	2	4.5	1	1	+	No	None	Yes	Bulk	No
JIA 36	PB, SF	ERA	Persistent	ERA	F	13	13.5	1	1	-	No	MTX	No	Bulk	No
JIA 37	SF	Oligo	Persistent	Persistent	F	2.5	8	1	2	-	Yes	MTX	No	No	Suppr assay
JIA 38	SF	Oligo	Persistent	Persistent	F	3	7.5	1	2	?	No	None	No	No	Suppr assay

NA, not applicable; PB, peripheral blood; SF, synovial fluid; Oligo, oligoarticular; ERA, enthesitis-related arthritis; JIA, juvenile idiopathic arthritis; F, female; M, male; yrs, years; ADA, adalimumab; ETA, etanercept; LEF, leflunomide; MTX, methotrexate; ANA, autonuclear antibodies; Suppr, suppression. *jointly with systemic steroids.

Table S2: Antibodies and dyes used in flow cytometry staining.

Antibody or dye	Clone	Manufacturer	Catalog #	Staining
anti-CD4-FITC	RPA-T4	BD Biosciences	555346	Surface
anti-CD25-PE	BC96	eBioscience	12-0259-42	Surface
anti-CD127-PE/Cy7	A019D5	BioLegend	351320	Surface
anti-CD45RO-FITC	UCHL1	BioLegend	304204	Surface
anti-CD3-APC	UCHT1	eBioscience	17-0038-42	Surface
anti-CD3-PE/Cy7	OKT3	BioLegend	317334	Surface
anti-CD4-PE	OKT4	BioLegend	317410	Surface
anti-CD8-APC/Cy7	RPA-T8	BioLegend	301015	Surface
anti-Tgd-FITC	B1.1	BioLegend	11-9959-42	Surface
anti-CXCR3-Pacific Blue	G025H7	BioLegend	353724	Surface
anti-CXCR3-BV605	G025H7	BioLegend	353728	Surface
anti-CD161-BV605	HP-3G10	BioLegend	339916	Surface
anti-CTLA4-APC	L3D10	BioLegend	349908	Intracellular
anti-Helios-PerCp/Cy5.5	22F6	BioLegend	137230	Intracellular
anti-Helios-APC/eFluor780	22F6	eBioscience	47-9883-42	Intracellular
anti-FOXP3-pacific blue	PCH101	Invitrogen	48-4776-42	Intracellular
anti-IFNγ-APC	4S.B3	BioLegend	502512	Intracellular
anti-IL-17A-PerCp/Cy5.5	BL168	BioLegend	512314	Intracellular
LIVE/DEAD^{TM} Fixable Yellow Dead Cell Stain	NA	Invitrogen	L34959	Dead cells

Table S3: Location of CpG sites analyzed in methylations studies.

Locus	Region	Position*	CpG count
FOXP3	Treg-specific demethylation region (CNS2 region)	-2330 to -2263	9
CTLA4	Exon 2	2651 to 2757	5
IKZF2	Intron 4	27732 to 27748	3
IKZF4	Intron 5	1969 to 2083	2
IL2RA	Intron 1	24448 to 24517	4

* Positions are specified counting from the ATG translation start codon.

Table S4: Significant hits in independent Gene Set Enrichment Analyses (GSEA) of SF Tregs versus PB Tregs and SF Teffs versus PB Teffs. Tregs ($CD4^{+}CD25^{+}CD127^{lo}$) and Teffs ($CD4^{+}CD25^{-}$) were sorted from paired SF and PB samples of JIA patients (n=14) and from the PB of pediatric (n=5) and adult (n=4) controls for bulk RNA-sequencing. The normalized enrichment score (NES), adjusted p-value and q-values as estimated by clusterProfiler are reported for significantly enriched pathways or gene sets, ranked from highest to lowest NES in the SF Treg compartment. DESeq2-estimated and rank-corrected log2 fold changes (LFC) in gene expression values served as input for a cutoff-free GSEA, run in R with clusterProfiler version 3.14.3. GSEA compared sequencing data to the Reactome database (immune system and metabolism subsets), the human Treg gene set described in Ferraro (2014) and the mouse peripheral/induced Treg gene set described in Hill (2007). n.s., not significant.

Pathway ID	Pathway or Gene set description	NES (Teff)	NES (Treg)	P-value (Teff)	P-value (Treg)	Q-values (Teff)	Q-values (Treg)
Treg_UP	Treg signature (Ferraro, 2014) - upregulated genes	2.734	2.538	0.00329	0.00297	0.00265	0.00207
R-HSA-6783783	Interleukin-10 signaling	2.325	2.532	0.00329	0.00297	0.00265	0.00207
R-HSA-2132295	MHC class II antigen presentation	2.092	2.505	0.00329	0.00297	0.00265	0.00207
R-HSA-877300	Interferon gamma signaling	2.162	2.503	0.00329	0.00297	0.00265	0.00207
R-HSA-909733	Interferon alpha/beta signaling	2.002	2.402	0.00329	0.00297	0.00265	0.00207
R-HSA-913531	Interferon Signaling	2.115	2.357	0.00329	0.00297	0.00265	0.00207
R-HSA-1236978	Cross-presentation of soluble exogenous antigens (endosomes)	n.s.	2.277	n.s.	0.00297	n.s.	0.00207
R-HSA-350562	Regulation of ornithine decarboxylase (ODC)	n.s.	2.269	n.s.	0.00297	n.s.	0.00207
R-HSA-389948	PD-1 signaling	2.264	2.234	0.00329	0.00297	0.00265	0.00207
R-HSA-6798695	Neutrophil degranulation	1.932	2.227	0.00329	0.00297	0.00265	0.00207
R-HSA-202427	Phosphorylation of CD3 and TCR zeta chains	2.209	2.192	0.00329	0.00297	0.00265	0.00207
R-HSA-449147	Signaling by Interleukins	1.987	2.164	0.00329	0.00297	0.00265	0.00207
R-HSA-202430	Translocation of ZAP-70 to Immunological synapse	2.175	2.134	0.00329	0.00297	0.00265	0.00207
R-HSA-202424	Downstream TCR signaling	1.585	2.106	0.02621	0.00297	0.02113	0.00207
R-HSA-202403	TCR signaling	1.508	2.081	0.04549	0.00297	0.03667	0.00207
R-HSA-202433	Generation of second messenger molecules	1.750	2.065	0.03377	0.00297	0.02722	0.00207
R-HSA-5668541	TNFR2 non-canonical NF-kB pathway	n.s.	2.046	n.s.	0.00297	n.s.	0.00207
R-HSA-388841	Costimulation by the CD28 family	1.967	2.029	0.00329	0.00297	0.00265	0.00207
R-HSA-351202	Metabolism of polyamines	n.s.	2.001	n.s.	0.00297	n.s.	0.00207
R-HSA-5669034	TNFs bind their physiological receptors	n.s.	1.995	n.s.	0.00297	n.s.	0.00207
R-HSA-1236974	ER-Phagosome pathway	n.s.	1.925	n.s.	0.00297	n.s.	0.00207
R-HSA-6785807	Interleukin-4 and Interleukin-13 signaling	1.543	1.916	0.04465	0.00297	0.03599	0.00207
R-HSA-1236975	Antigen processing-Cross presentation	n.s.	1.877	n.s.	0.00443	n.s.	0.00308
R-HSA-446652	Interleukin-1 family signaling	1.514	1.821	0.03886	0.00297	0.03133	0.00207
R-HSA-1169091	Activation of NF-kappaB in B cells	n.s.	1.821	n.s.	0.00894	n.s.	0.00622
R-HSA-2160916	Hyaluronan uptake and degradation	n.s.	1.800	n.s.	0.02123	n.s.	0.01477

R-HSA-193368	Synthesis of bile acids and bile salts via 7alpha-hydroxycholesterol	n.s.	1.795	n.s.	0.02333	n.s.	0.01623
R-HSA-198933	Immunoregulatory interactions between a Lymphoid and a non-Lymphoid cell	1.686	1.789	0.00329	0.00443	0.00265	0.00308
R-HSA-622312	Inflammasomes	n.s.	1.788	n.s.	0.02368	n.s.	0.01647
R-HSA-1168372	Downstream signaling events of B Cell Receptor (BCR)	n.s.	1.785	n.s.	0.00894	n.s.	0.00622
R-HSA-447115	Interleukin-12 family signaling	1.678	1.761	0.03245	0.01554	0.02616	0.01081
R-HSA-9020591	Interleukin-12 signaling	1.831	1.747	0.00828	0.02367	0.00667	0.01646
R-HSA-9020702	Interleukin-1 signaling	n.s.	1.745	n.s.	0.00621	n.s.	0.00432
R-HSA-5676590	NIK-->noncanonical NF-kB signaling	n.s.	1.742	n.s.	0.01554	n.s.	0.01081
R-HSA-449836	Other interleukin signaling	n.s.	1.729	n.s.	0.03297	n.s.	0.02293
R-HSA-918233	TRAF3-dependent IRF activation pathway	n.s.	1.726	n.s.	0.04085	n.s.	0.02842
R-HSA-197264	Nicotinamide salvaging	n.s.	1.724	n.s.	0.03665	n.s.	0.02549
R-HSA-193775	Synthesis of bile acids and bile salts via 24-hydroxycholesterol	n.s.	1.721	n.s.	0.04123	n.s.	0.02868
R-HSA-189445	Metabolism of porphyrins	n.s.	1.721	n.s.	0.03665	n.s.	0.02549
R-HSA-192105	Synthesis of bile acids and bile salts	n.s.	1.718	n.s.	0.03725	n.s.	0.02591
R-HSA-1630316	Glycosaminoglycan metabolism	n.s.	1.712	n.s.	0.00780	n.s.	0.00542
R-HSA-390918	Peroxisomal lipid metabolism	n.s.	1.706	n.s.	0.03725	n.s.	0.02591
R-HSA-77289	Mitochondrial Fatty Acid Beta-Oxidation	n.s.	1.703	n.s.	0.03297	n.s.	0.02293
R-HSA-6803157	Antimicrobial peptides	2.002	1.701	0.00329	0.04070	0.00265	0.02831
R-HSA-193807	Synthesis of bile acids and bile salts via 27-hydroxycholesterol	n.s.	1.701	n.s.	0.04800	n.s.	0.03339
R-HSA-933541	TRAF6 mediated IRF7 activation	n.s.	1.689	n.s.	0.04315	n.s.	0.03002
R-HSA-8956321	Nucleotide salvage	n.s.	1.689	n.s.	0.04315	n.s.	0.03002
R-HSA-211976	Endogenous sterols	n.s.	1.687	n.s.	0.04315	n.s.	0.03002
R-HSA-5607764	CLEC7A (Dectin-1) signaling	n.s.	1.680	n.s.	0.00780	n.s.	0.00542
pTreg_DN	induced/peripheral Treg (Hill, 2007) - downregulated genes	n.s.	1.662	n.s.	0.00297	n.s.	0.00207
R-HSA-8956319	Nucleobase catabolism	n.s.	1.655	n.s.	0.04845	n.s.	0.03370
R-HSA-2162123	Synthesis of Prostaglandins (PG) and Thromboxanes (TX)	n.s.	1.654	n.s.	0.06257	n.s.	0.04352
R-HSA-2871837	FCERI mediated NF-kB activation	n.s.	1.651	n.s.	0.01554	n.s.	0.01081
R-HSA-70263	Gluconeogenesis	n.s.	1.645	n.s.	0.05211	n.s.	0.03625
R-HSA-2142845	Hyaluronan metabolism	n.s.	1.642	n.s.	0.06086	n.s.	0.04234
R-HSA-2029482	Regulation of actin dynamics for phagocytic cup formation	n.s.	1.641	n.s.	0.02333	n.s.	0.01623
R-HSA-8978868	Fatty acid metabolism	n.s.	1.641	n.s.	0.00453	n.s.	0.00315
R-HSA-844456	The NLRP3 inflammasome	n.s.	1.636	n.s.	0.06257	n.s.	0.04352
R-HSA-15869	Metabolism of nucleotides	n.s.	1.620	n.s.	0.01554	n.s.	0.01081
R-HSA-428157	Sphingolipid metabolism	n.s.	1.611	n.s.	0.02207	n.s.	0.01535
R-HSA-5607761	Dectin-1 mediated noncanonical NF-kB signaling	n.s.	1.604	n.s.	0.03281	n.s.	0.02282

R-HSA-71387	Metabolism of carbohydrates	n.s.	1.586	n.s.	0.00297	n.s.	0.00207
R-HSA-983705	Signaling by the B Cell Receptor (BCR)	n.s.	1.584	n.s.	0.01408	n.s.	0.00980
R-HSA-5621481	C-type lectin receptors (CLRs)	n.s.	1.583	n.s.	0.01408	n.s.	0.00980
R-HSA-936440	Negative regulators of DDX58/IFIH1 signaling	n.s.	1.580	n.s.	0.06879	n.s.	0.04785
R-HSA-451927	Interleukin-2 family signaling	1.878	1.575	0.00582	0.05351	0.00469	0.03723
R-HSA-983169	Class I MHC mediated antigen processing & presentation	n.s.	1.530	n.s.	0.00297	n.s.	0.00207
R-HSA-211945	Phase I - Functionalization of compounds	n.s.	1.530	n.s.	0.05351	n.s.	0.03723
R-HSA-2029480	Fcgamma receptor (FCGR) dependent phagocytosis	n.s.	1.500	n.s.	0.04315	n.s.	0.03002
R-HSA-1169410	Antiviral mechanism by IFN-stimulated genes	n.s.	1.476	n.s.	0.05466	n.s.	0.03802
R-HSA-1428517	The citric acid (TCA) cycle and respiratory electron transport	n.s.	1.469	n.s.	0.02207	n.s.	0.01535
R-HSA-983168	Antigen processing: Ubiquitination & Proteasome degradation	n.s.	1.460	n.s.	0.00890	n.s.	0.00619
R-HSA-8957322	Metabolism of steroids	n.s.	1.459	n.s.	0.03794	n.s.	0.02639
R-HSA-1483257	Phospholipid metabolism	n.s.	1.390	n.s.	0.04315	n.s.	0.03002
R-HSA-71291	Metabolism of amino acids and derivatives	-2.205	-2.135	0.00329	0.00297	0.00265	0.00207
Treg_DN	Treg signature (Ferraro, 2014) - downregulated genes	-1.850	-2.277	0.00582	0.00297	0.00469	0.00207
R-HSA-2408522	Selenoamino acid metabolism	-2.974	-3.124	0.00329	0.00297	0.00265	0.00207
R-HSA-2408557	Selenocysteine synthesis	-3.089	-3.218	0.00329	0.00297	0.00265	0.00207
R-HSA-9008059	Interleukin-37 signaling	1.757	n.s.	0.04062	n.s.	0.03274	n.s.
R-HSA-196757	Metabolism of folate and pterines	-1.901	n.s.	0.00329	n.s.	0.00265	n.s.
